# Systematic Review of the Risk of Adverse Outcomes Associated with Vascular Endothelial Growth Factor Inhibitors for the Treatment of Cancer

**DOI:** 10.1371/journal.pone.0101145

**Published:** 2014-07-02

**Authors:** Labib Imran Faruque, Meng Lin, Marisa Battistella, Natasha Wiebe, Tony Reiman, Brenda Hemmelgarn, Chandra Thomas, Marcello Tonelli

**Affiliations:** 1 University of Alberta, Edmonton, Canada; 2 University of Toronto, Toronto, Canada; 3 Dalhousie University, Halifax, Canada; 4 University of Calgary, Calgary, Canada; Center for Interdisciplinary Research in Biology (CIRB) is a novel Collège de France/CNRS/INSERM, France

## Abstract

**Background:**

Anti-angiogenic therapy targeted at vascular endothelial growth factor (VEGF) is now used to treat several types of cancer. We did a systematic review of randomized controlled trials (RCTs) to summarize the adverse effects of vascular endothelial growth factor inhibitors (VEGFi), focusing on those with vascular pathogenesis.

**Methods and Findings:**

We searched MEDLINE, EMBASE and Cochrane Library until April 19, 2012 to identify parallel RCTs comparing a VEGFi with a control among adults with any cancer. We pooled the risk of mortality, vascular events (myocardial infarction, stroke, heart failure, and thromboembolism), hypertension and new proteinuria using random-effects models and calculated unadjusted relative risk (RR). We also did meta-regression and assessed publication bias. We retrieved 83 comparisons from 72 studies (n = 38,078) on 11 different VEGFi from 7901 identified citations. The risk of mortality was significantly lower among VEGFi recipients than controls (pooled RR 0.96, 95% confidence interval [CI] 0.94 to 0.98, I^2^ = 0%, tau2 = 0; risk difference 2%). Compared to controls, VEGFi recipients had significantly higher risk of myocardial infarction (MI) (RR 3.54, 95% CI 1.61 to 7.80, I^2^ = 0%, tau2 = 0), arterial thrombotic events (RR 1.80, 95% CI 1.24 to 2.59, I^2^ = 0%, tau2 = 0); hypertension (RR 3.46, 95% CI 2.89 to 4.15, I^2^ = 58%, tau2 = 0.16), and new proteinuria (RR 2.51, 95% CI 1.60 to 3.94, I^2^ = 87%, tau2 = 0.65). The absolute risk difference was 0.8% for MI, 1% for arterial thrombotic events, 15% for hypertension and 12% for new proteinuria. Meta-regression did not suggest any statistically significant modifiers of the association between VEGFi treatment and any of the vascular events. Limitations include heterogeneity across the trials.

**Conclusions:**

VEGFi increases the risk of MI, hypertension, arterial thromboembolism and proteinuria. The absolute magnitude of the excess risk appears clinically relevant, as the number needed to harm ranges from 7 to 125. These adverse events must be weighed against the lower mortality associated with VEGFi treatment.

## Introduction

Angiogenesis is essential for tumour growth and blood borne metastasis [1], and vascular endothelial growth factor (VEGF) plays a key role in angiogenesis as well as the phenotyping of blood vessels in tumors [2]. Anti-angiogenic therapy targeted at VEGF inhibits vascular growth affecting the survival of certain tumor cells and has specificity through expression of specific markers by activated endothelium. Other mechanisms may also be important – such as improving blood perfusion, oxygenation or drug delivery [3]–[6]. Two major approaches for disrupting VEGF signaling include ligand blockade and pharmacologic inhibition. Ligand could be blocked through a monoclonal antibody (MoAb), soluble receptor/ligand trap, or an aptamer and signaling is inhibited by receptor targeting using a MoAb or a small-molecule tyrosine kinase (TK) inhibitor [7].

Several VEGF inhibitors (VEGFi) have been approved by the Food and Drug Administration (FDA) for use in the treatment of cancer, beginning with bevacizumab for metastatic colorectal cancer in 2004 [1]. VEGFi are now used to treat multiple other types of cancer including lung adenocarcinoma, advanced renal cell carcinoma, gastrointestinal stromal tumor and medullary thyroid cancer. Although they have potentially important clinical benefits, VEGFi can also cause dose-dependent and dose-independent vascular adverse reactions [1], [2], [7], [8]. FDA withdrew its approval of bevacizumab for breast cancer treatment in 2011, considering that the risk of such treatment would outweigh its benefits [9]–[12]. Given the mechanism of action for VEGFi, hypertension and ischemic coronary and cerebrovascular events have been of particular concern. Although arterial thrombosis, venous thrombosis, and compromise of vascular organs such as the kidney are also of potential concern, these adverse outcomes have been less well studied. We did this systematic review and meta-analysis to summarize available randomized trial evidence on the adverse effects of vascular endothelial growth factor inhibitors compared to control. Given the mechanism of action for VEGFi, we focused on adverse events that are related to vascular disease (myocardial infarction, stroke, heart failure, hypertension, thromboembolism, and proteinuria).

## Methods

We did a systematic review and meta-analysis of published randomized clinical trials. We used accepted methods for literature searches, article selection, data extraction and risk of bias assessment and have reported our results according to published guidelines [13].

### Data sources and searches

An expert librarian did a comprehensive search to identify all relevant studies regardless of language or publication status. MEDLINE (1950- April 19, 2012), EMBASE (1980- April 19, 2012) and Cochrane Library (April 19, 2012) were searched. The full search strategies are available in eTable S1. An academic subject-specialist and a statistician screened each citation or abstract. Trials considered to be relevant by any reviewer were retrieved for further review.

### Intervention and comparison

VEGF inhibitor functions with a monoclonal IgG1 antibody against VEGF (such as bevacizumab); typical VEGF receptor inhibitor inhibits VEGF receptors on cancer cells (such as a tyrosine kinase inhibitor sunitinib) and atypical VEGF receptor inhibitor includes drugs having multikinase inhibitor properties such as sorafenib which inhibits VEGF receptors and the Raf cascade. A list of eligible VEGFi agents is shown in eTable S2. We compared VEGFi therapy to placebo (or no active intervention). Cointervention was allowed in both intervention and control arms.

### Study selection

The full text of each potentially relevant study was independently assessed by two reviewers for inclusion in the review using predetermined eligibility criteria on a printed form. Parallel RCTs were eligible for inclusion if they involved adults (16 years or older) with cancer and included at least 30 participants in each treatment group; they compared a VEGFi with a control (placebo or no active treatment); and they reported one or more clinical outcomes (mortality, cardiovascular events [myocardial infarction, stroke, heart failure or hypertension], new proteinuria or thromboembolic events). The primary outcome measure was all-cause mortality. We excluded studies published in languages other than English; crossover studies were eligible but only results before the crossover were included. Disagreements were resolved by discussion and consultation with a third party. Disagreements arose with 4% of the articles (kappa = 0.90).

### Data extraction and risk of bias assessment

We assessed and reported risks of bias in included studies using items from the Chalmers index (intention-to-treat, method of handling missing data) as well as items (concealment of allocation, randomization, blinding, loss-to-follow-up, funding sources, early stopping) that have been shown empirically to affect internal validity [14]–[18]. The following properties were extracted from each study: characteristics (country, VEGFi type and dose, duration of follow-up, duration of treatment, study cointervention(s), incident vs prevalent population [based on whether the index cancer had been previously treated or not], sample size), participants (age, gender, cancer type and stage, number of organs with metastases, prior chemotherapy or radiotherapy, Eastern Cooperative Oncology Group [ECOG] performance status), and results (number of events for subgroups, unadjusted and adjusted HR for eligible outcomes). The following outcomes were considered: all-cause mortality, cardiovascular events (myocardial infarction, stroke, heart failure, and hypertension), thrombosis (thrombotic/thromboembolic events, arterial thrombotic/thromboembolic events, venous thrombotic/thromboembolic events, and pulmonary embolism), and new proteinuria at the end of study. For each study, we used the definition of each outcome as provided by the authors of the source publication.

One reviewer extracted data from the selected trials. A second reviewer checked for accuracy. We preferentially captured intention-to-treat analyses where presented. Disagreements were resolved with the aid of a third party.

### Data synthesis and analysis

We used Stata MP software (www.stata.com) to pool results using random-effects models. Dichotomous outcomes were summarized using the unadjusted relative risk (RR) and statistical heterogeneity was quantified using the I^2^ statistic. We also used univariable meta-regression to examine whether certain variables (median age, percentage of male participants, VEGFi type, median duration of follow-up, median duration of treatment, incident population, cancer type, percentage of participants in cancer stages, number of organs with metastases, prior chemotherapy and radiotherapy, ECOG performance status, and study risks of bias) influenced the association between VEGFi therapy and clinical outcome. We used random effect meta-regression. Log-RR was used as a summary statistics for the dependent variable. Publication bias was assessed by using weighted regression of data from trials that reported the frequency of the primary outcome by treatment group.

## Results

### Quantity of research available

**Figure 1:**
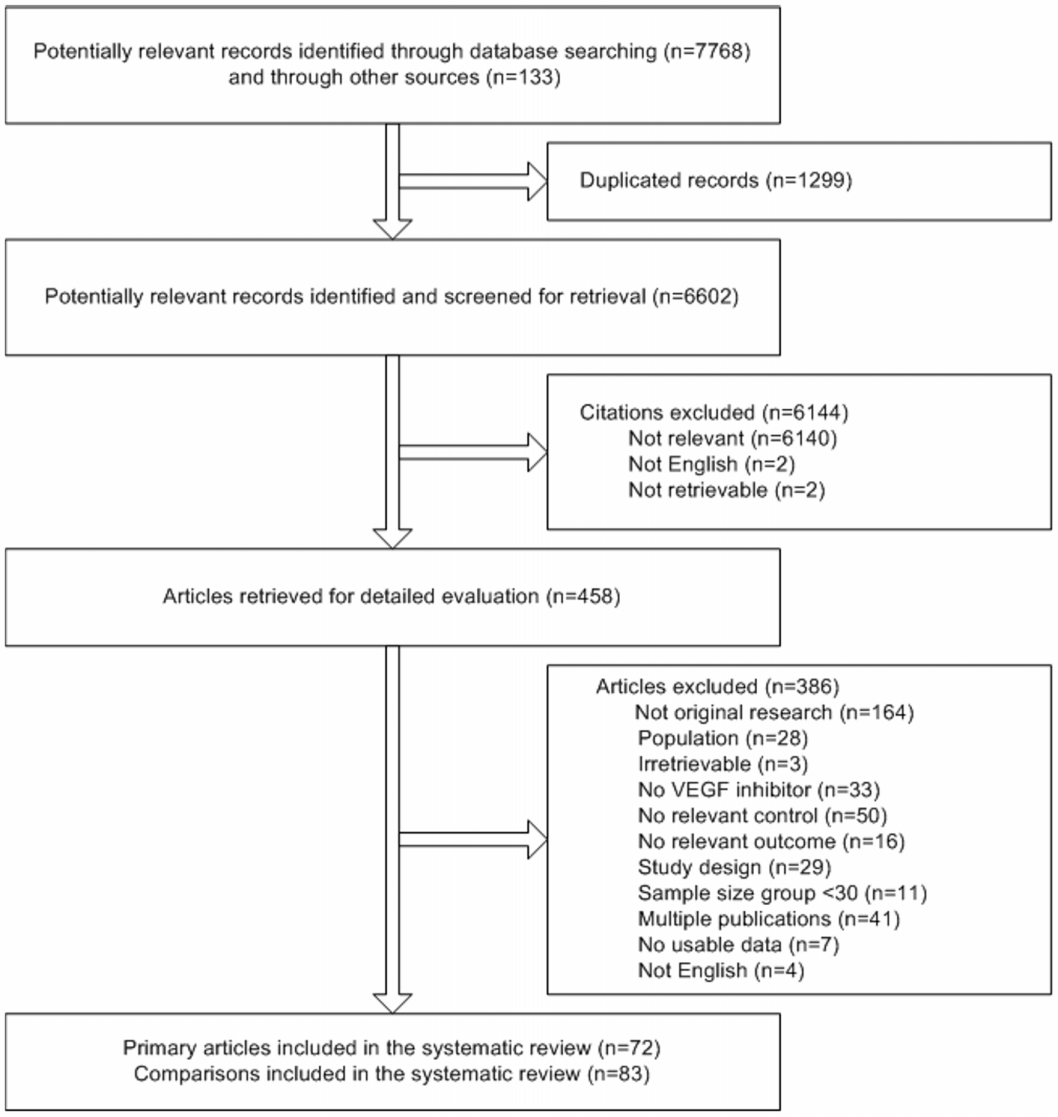
PRISMA flow diagram.

From 7901 identified citations, 458 articles were retrieved for detailed evaluation (Figure 1). Of these, 83 comparisons from 72 studies (n = 38,078) were eligible for inclusion in this review (Table 1 and eReference S1). Study sample sizes ranged from 61 to 2,670 (median 331); the median duration of treatment was 18 (range 3–90) weeks; the median duration of post-treatment follow-up was 15 (range 6–44) months. Details of the studies are summarized in eTable S3.

**Table 1:** Brief description of included randomized trials.

author	year	Country	type of tumour/macular degeneration	VEGFi	cointervention	VEGFi weekly dose	sample size
Kabbinavar	2003	USA	metastatic colorectal cancer	bevacizumab	FU/LV	2.5; 5 mg/kg	104
Yang	2003	USA	metastatic RCC	bevacizumab	none	1.5; 5 mg/kg	116
Hurwitz	2004	USA, New Zealand, Australia	metastatic colorectal cancer	bevacizumab	IFL	2.5 mg/kg	813
Johnson	2004	USA	NSCLC	bevacizumab	carboplatin & paclitaxel	2.5; 5 mg/kg	99
Kabbinavar	2005	USA	metastatic colorectal cancer	bevacizumab	FU/LV	2.5 mg/kg	209
Miller	2005	USA	metastatic breast cancer	bevacizumab	capecitabine	5 mg/kg	462
Demetri	2006	USA, Canada, Australia, Italy, Singapore, UK, Belgium, France, Netherlands	gastrointestinal stromal tumour	sunitinib	none	350 mg	312
Ratain	2006	USA, UK	RCC	sorafenib	none	5600 mg	65
Sandler	2006	USA	NSCLC	bevacizumab	paclitaxel/carboploatin	5 mg/kg	850
Arnold	2007	Canada	SCLC	vandetanib	none	2100 mg	107
Cohen	2007	USA	NSCLC	bevacizumab	carboplatin & paclitaxel	5 mg/kg	878
Escudier*	2007	France, USA, Poland, Canada	metastatic RCC	Neovastat	none	1680 ml	300
Escudier*	2007	France, USA, UK, Poland, Germany	metastatic RCC	sorafenib	none	5600 mg	903
**author**	**year**	**country**	**type of tumour/macular degeneration**	**VEGFi**	**cointervention**	**VEGFi weekly dose**	**sample size**
Giantonio$	2007	USA, South Africa	metastatic colorectal cancer	bevacizumab	FOLFOX4	5 mg/kg	432
Herbst$	2007	USA	NSCLC	bevacizumab	docetaxel/pemetrexed	5 mg/kg	61
Heymach	2007	USA, Czech Republic, Hungary	NSCLC	vandetanib	docetaxel	700; 2100 mg	127
Karrison	2007	USA	malignant mesothelioma	bevacizumab	gemcitabine & cisplatin	5 mg/kg	108
Mao	2007	USA	prostate cancer	IM 862	none	70 mg	71
Miller	2007	USA, Canada	metastatic breast cancer	bevacizumab	paclitaxel	5 mg/kg	673
Heymach$	2008	USA, Spain, German, India, South Africa	NSCLC	vandetanib	paclitaxel & carboplatin	2100 mg	86
Llovet	2008	Europe, Australia, North America, South America	hepatocellular carcinoma	sorafenib	none	5600 mg	602
McDermott	2008	USA	melanoma	sorafenib	dacarbazine	5600 mg	101
Saltz	2008	USA, Canada, UK, Australia, Spain, Austria, Taiwan, Switzerland	metastatic colorectal cancer	bevacizumab	XELOX or FOLFOX-4	2.5 mg/kg	1400
Spano	2008	France, Spain, UK, Canada, USA, Italy	pancreatic cancer	axitinib	gemcitabine	70 mg	103
Allegra	2009	USA, Ireland	colon cancer	bevacizumab	FOLFOX	2.5 mg/kg	2670
Cheng	2009	Taiwan, China, South Korea	hepatocellular carcinoma	sorafenib	none	5600 mg	226
Hauschild	2009	Germany, USA, France, Canada, Australia, UK, Netherlands	melanoma	sorafenib	carboplatin/paclitaxel	5600 mg	270
**author**	**year**	**country**	**type of tumour/macular degeneration**	**VEGFi**	**cointervention**	**VEGFi weekly dose**	**sample size**
Horti	2009	Hungary, Germany, Brazil, Sweden, South Africa	metastatic prostate cancer	vandetanib	docetaxel & prednisolone	700 mg	86
Van Cutsem	2009	Belgium, France, Canada, Netherlands, Austria, Switzerland	metastatic pancreatic cancer	bevacizumab	gemcitabine & erlotinib	2.5 mg/kg	607
Abou-Alfa	2010	USA, UK, Canada, Russia, Argentina, China	hepatocellular carcinoma	sorafenib	doxorubicin	5600 mg	96
Crown	2010	Ireland, France, UK, Poland, USA	breast cancer	sunitinib	capecitabine	262.5 mg	442
Escudier	2010	Europe, Australia, Israel, Singapore, Taiwan	metastatic RCC	bevacizumab	interferon alfa-2a	5 mg/kg	649
Goss	2010	Canada, Brazil, Argentina, Romania, Australia, Singapore	NSCLC	cediranib	carboplatin/paclitaxel	210 mg	251
Herbst	2010	USA, China, Germany, Belgium, Japan, Netherlands	NSCLC	vandetanib	docetaxel	700 mg	1391
Kemeny	2010	USA	metastatic colorectal adenocarcinoma	bevacizumab	HAI with irinotecan or oxaliplatin/fluorouracil/leucovorin	2.5 mg/kg	73
Kindler	2010	USA	pancreatic cancer	bevacizumab	gemcitabine	5 mg/kg	602
Lu	2010	USA, Canada	NSCLC	Neovastat	paclitaxel & carboplatin, or cisplatin & vinorelbine	1680 ml	379
Miles	2010	UK, Australia, Canada, South Korea, Europe	breast cancer	bevacizumab	docetaxel	2.5; 5 mg/kg	736
**author**	**year**	**country**	**type of tumour/macular degeneration**	**VEGFi**	**cointervention**	**VEGFi weekly dose**	**sample size**
Monk	2010	USA, Peru, Argentina, Spain, France, Thailand	cervical cancer	pazopanib	lapatinib	5600 mg	115
Reck	2010	Germany, Czech Republic, Poland, Canada, Russia, Switzerland, UK	NSCLC	bevacizumab	gemcitabine & cisplatin	2.5; 5 mg/kg	1043
Rini	2010	USA, Canada	metastatic RCC	bevacizumab	interferon alfa	5 mg/kg	732
Scagliotti	2010	Italy, Germany, Hungary, Poland, Brazil, Chile, USA	NSCLC	sorafenib	carboplatin/paclitaxel	5600 mg	926
Serve	2010	Germany	acute myeloid leukemia	sorafenib	standard induction chemotherapy+ consolidation therapy	5600 mg	197
Stathopoulos	2010	Greece	colorectal cancer	bevacizumab	irinotecan, 5-FU, leucovorin	2.5 mg/kg	222
Sternberg	2010	Australia, New Zealand, South Korea, Europe, South America	RCC	pazopanib	none	5600 mg	435
Tebbutt	2010	Australia, New Zealand, USA	metastatic colorectal adenocarcinoma	bevacizumab	capecitabine	2.5 mg/kg	235
Brufsky	2011	USA	metastatic breast cancer	bevacizumab	taxane/gemcitabine/capecitabine/vinorelbine	5 mg/kg	684
Burger	2011	USA, Canada, South Korea, Japan	ovarian cancer	bevacizumab	paclitaxel & carboploatin	5 mg/kg	1873
Choueiri	2011	USA	urothelial cancer	vandetanib	docetaxel	700 mg	142
de Boer	2011	Belgium, Australia, Mexico, UK, Philippines, South Africa, Italy, Germany, Taiwan	NSCLC	vandetanib	pemetrexed	700 mg	534
**author**	**year**	**country**	**type of tumour/macular degeneration**	**VEGFi**	**cointervention**	**VEGFi weekly dose**	**sample size**
Guan	2011	China	metastatic colorectal cancer	bevacizumab	irinotecan/5-FU/leucovorin	2.5 mg/kg	214
Hecht	2011	USA, Germany, Canada, Hungary, Finland, Qatar	metastatic colorectal adenocarcinoma	PTK/ZK	FOLFOX 4	8750 mg	1168
Herbst	2011	USA	NSCLC	bevacizumab	erlotinib	5 mg/kg	636
Kato	2011	Japan	colorectal cancer	cediranib	FOLFOX6	140; 210 mg	172
Kim	2011	USA, Switzerland	melanoma	bevacizumab	paclitaxel & carboploatin	5 mg/kg	214
Kindler	2011	USA, Japan, Netherlands, France, Canada, South Korea, UK, Belgium	pancreatic cancer	axitinib	gemcitabine	70 mg	630
Kudo	2011	Japan, South Korea	hepatocellular carcinoma	sorafenib	none	5600 mg	458
Loriot	2011	France	metastatic pancreatic cancer	vandetanib	bicalutamide	2100 mg	95
Martin	2011 A	Spain, France, Hungary, Ireland, Canada, Germany, India, Poland, USA	breast cancer	motesanib	paclitaxel	875 mg	138
Martin	2011 B	Spain, France, Hungary, Ireland, Canada, Germany, India, Poland, USA	breast cancer	bevacizumab	paclitaxel	5 mg/kg	144
Ohtsu	2011	Japan, South Korea, Europe, Pan-America	gastric cancer	bevacizumab	cisplatin & capecitabine/FU	2.5 mg/kg	774
Perren	2011	UK, Germany, Canada, France, Finland, Australia, Norway, Spain, Denmark, Sweden	ovarian cancer	bevacizumab	paclitaxel & carboploatin	2.5 mg/kg	1528
Raymond	2011	France, South Korea, UK, Canada, Taiwan, Germany, USA	pancreatic neuroendocrine tumor	sunitinib	none	262.5 mg	171
**author**	**year**	**country**	**type of tumour/macular degeneration**	**VEGFi**	**cointervention**	**VEGFi weekly dose**	**sample size**
Robert	2011 A	USA, France, UK, Ukraine, Russia	breast cancer	bevacizumab	capecitabine	5 mg/kg	615
Robert	2011 B	USA, France, UK, Ukraine, Russia	breast cancer	bevacizumab	taxane-based/anthracycline-based	5 mg/kg	622
Rugo	2011	USA, Spain, Canada, Italy, Germany, UK, India, Czech Republic	breast cancer	axitinib	docetaxel	70 mg	168
Spigel*	2011	USA	SCLC	bevacizumab	etoposide + cisplatin/carboplatin	5 mg/kg	102
Spigel*	2011	USA	NSCLC	sorafenib	erlotinib	5600 mg	166
Van Cutsem	2011	Belgium, Italy, UK, Germany, Canada, USA	metastatic colorectal adenocarcinoma	PTK/ZK	FOLFOX 4	8750 mg	855
Wells Jr	2011	USA, Australia, Germany, Italy, France, Poland, UK	medullary thyroid cancer	vandetanib	none	2100 mg	331
Yang	2011	China	hepatocellular carcinoma	sorafenib	cryotherapy	5600 mg	102
Bear	2012	USA, Canada	breast cancer	bevacizumab	docetaxel/docetaxel-capecitabine/docetaxel-gemcitabine	5 mg/kg	1206
Kelly	2012	USA	metastatic prostate cancer	bevacizumab	docetaxel & prednisone	5 mg/kg	1050
von Minckwitz	2012	Germany, Switzerland	breast cancer	bevacizumab	docetaxel + epirubicin-cyclophoshamide	5 mg/kg	1925

NSCLC: non-small-cell lung cancer; RCC: renal cell carcinoma; SCLC: small-cell lung cancer; FOLFOX 4: oxaliplatin, leucovorin, fluorouracil; FU/LV:fluorouracil & leucovorin; IFL: irinotecan, bolus fluorouracil, leucovorin; XELOX: capecitabine & oxaliplatin; HAI Hepatic arterial infusion

$ Comparison between another arm and control was not eligible.

There are 74 rows for 72 studies reported in text: two studies, Martin 2011 had two arms Motesanib and bevacizumab comparing with placebo, Robert 2011 had two arms of capecitabine and taxane-based/anthracycline-based comparing bevacizumab versus placebo in each arm.

*These are two different trials published in the same year by the same author: Spigel et al in 2011 used bevacizumab for extensive stage small cell lung cancer in one trial and sorafenib for advanced non–small-cell lung cancer in another trial. Likewise, Escudier et al in 2007 used neovastat in metastatic renal cell carcinoma in one trial and sorafenib in advanced clear-cell renal-cell carcinoma in another trial.

### Risk of bias

**Figure 2:**
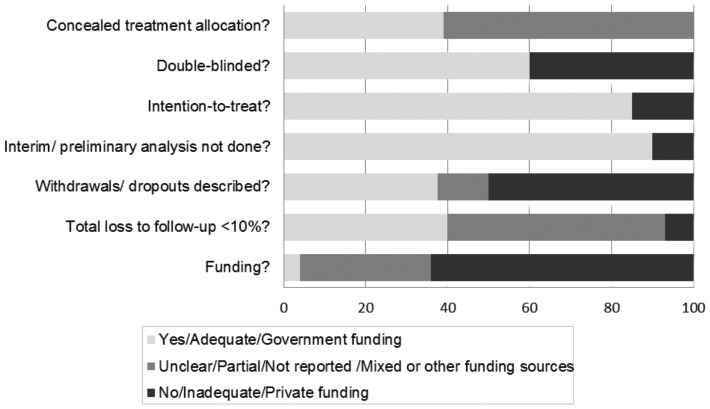
Risk of bias of included studies. The responses for each question in this risk of bias tool are represented by different colors, segmented along a horizontal bar. Light gray depicts the percent of studies responding with the smallest risk of bias. Medium gray depicts the percent of studies responding with a moderate or unclear risk of bias. Dark gray indicates the greatest risk of bias. The responses to “Concealed treatment allocation?” are adequate, inadequate and unclear. The responses to “Double-blinded?”, “Intention-to-treat?” and “Interim/preliminary analysis not done?” are yes, unclear and no. The responses to “Withdrawals/dropouts described” are yes, no or partial. The responses to “Total lost to follow up <10%” are yes, no or not reported. The responses to “Funding?” are government, private or mixed/other funding sources.

The 72 studies had generally moderate to high risks of bias (see Figure 2 and eTable S4). The method of randomization was inappropriate or not reported in 68% of studies; 61% did not adequately conceal treatment allocation. Forty percent did not describe their study as double-blind (28% did not report that participants were blinded to their treatment). Only 38% fully reported losses to follow-up. Most (86%) were industry sponsored or partially industry sponsored trials. On the other hand, most trials exhibited certain markers of high quality (90% did not stop their study early and 85% used an intention-to-treat approach). We found no evidence of publication bias for all-cause mortality using a weighted regression test (bias = −0.34, p = 0.20; see eFigure S1).

### Characteristics of trials and their participants

Of the eligible trials, 36 used a VEGF inhibitor (bevacizumab); 8 studies used a typical VEGF receptor inhibitor (axitinib in 3 trials, sunitinib in 3 trials, and cediranib in 2 trials); 29 trials used an atypical VEGF receptor inhibitor (vandetanib in 9 trials, sorafenib in 12 trials, vatalanib [hereafter referred to as its more commonly used name PTK/ZK] in 2 trials, pazopanib in 2 trials, neovastat in 2 trials, IM 862 in 1 trial, and motesanib in 1 trial, respectively). One trial had two active treatment arms (bevacizumab and motesanib) that were compared to placebo. Thirteen trials compared VEGFi therapy to placebo without any cointervention; the remainder included some type of chemotherapy cointerventions such as capecitabine, docetaxel or gemcitabine. The median age of study participants was 60 (range 48–71) years; the majority of patients were male (median 60%). Some studies reported cancer stage, ECOG performance status, previous chemotherapy or radiation therapy, and number of sites with metastases among study participants (see eTable S3).

### Mortality

**Figure 3:**
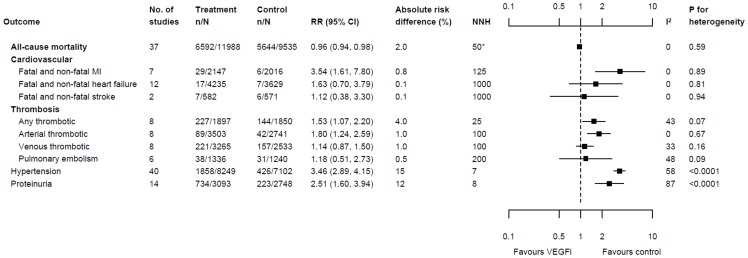
Pooled effect of treatment with VEGFi on clinical outcomes. *For all-cause mortality, the value presented is number needed to treat (NNT); however for all other outcomes number needed to harm (NNH) is presented. # 8 trials presented either only any thrombotic events or specified arterial and/or venous thromosis and/or pulmonary embolism in addition to any thrombotic events; therefore the included trials (in figure 4) and number of participants for any thrombotic events differed from the arterial, venous thrombosis or pulmonary embolism events.

Thirty-seven trials (44 comparisons; n = 21,523) reported frequency of all-cause mortality at the end of study. Mortality was significantly lower among participants in the VEGFi treatment groups than in the control groups (RR 0.96, 95% confidence interval [CI] 0.94 to 0.98, I^2^ = 0%, tau2 = 0; see Figure 3); this corresponded to a risk difference of 2% (risk of death was 59% among participants in the control groups) and number needed to treat of 50.

Except for the presence of cointervention administered during the study (RR for trials with cointervention 0.97, 95% CI 0.95 to 0.99, I^2^ = 0%, tau2 = 0; RR for trials without cointervention 0.82, 95% CI 0.70 to 0.94, I^2^ = 19%, tau2 = 0.006, p = 0.007 for difference), none of the covariates considered (see methods) significantly modified the association between VEGFi treatment and the risk of mortality in meta-regression (all p>0.08). In addition, there was no evidence from meta-regression that study risks of bias modified the association between VEGFi treatment and the risk of mortality (see eTable S5).

### Cardiovascular events

**Figure 4:**
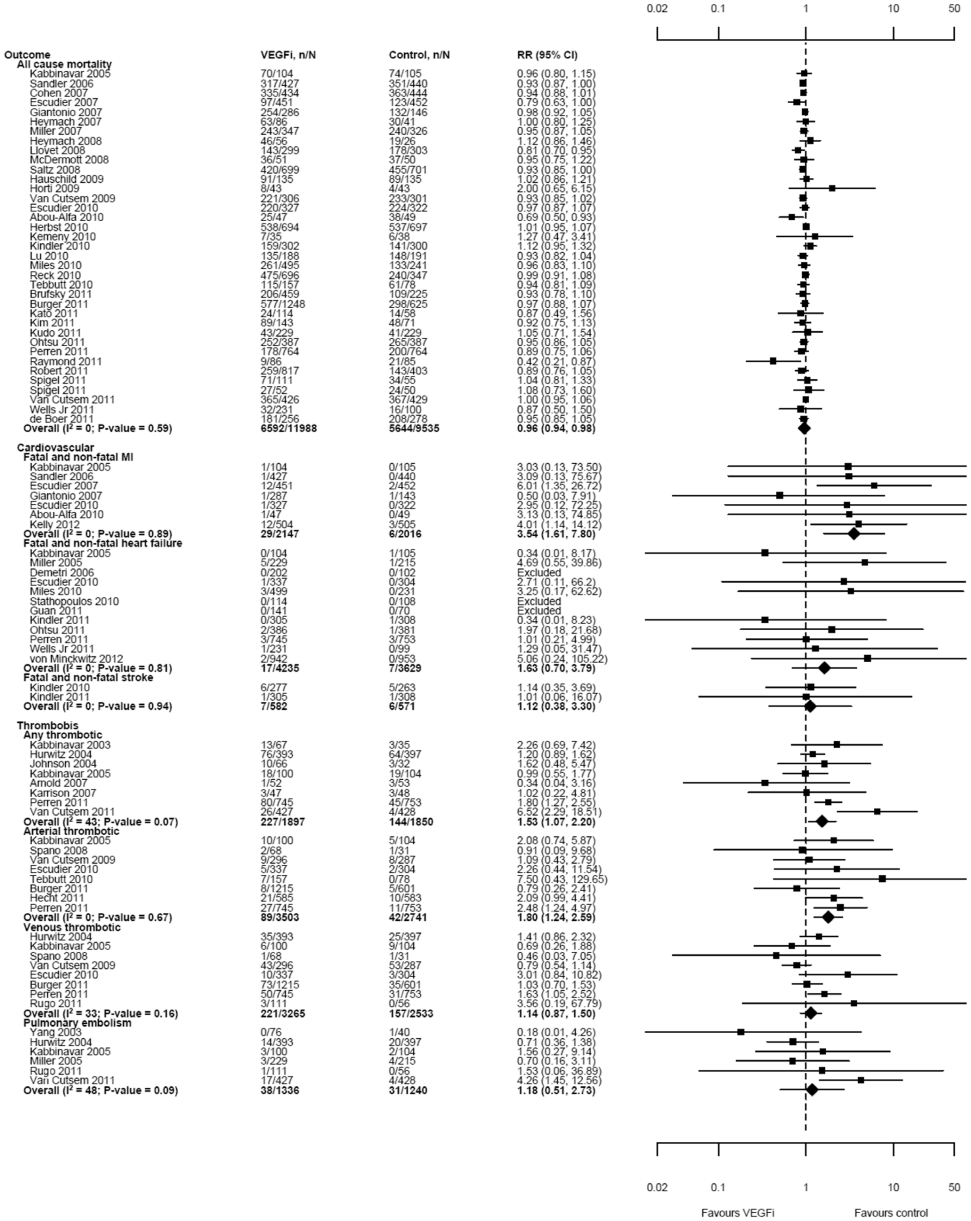
Effect of treatment with VEGFi on all-cause mortality, cardiovascular events and thrombosis.

Seven trials (n = 4,163), twelve trials (n = 7,864) and two trials (n = 1,153) reported the frequency of fatal or non-fatal myocardial infarction, heart failure and stroke, respectively. The pooled risk among VEGFi recipients was significantly higher for myocardial infarction, but not for heart failure or stroke (RR for myocardial infarction 3.54, 95% CI 1.61 to 7.80, I^2^ = 0%, tau2 = 0; RR for heart failure 1.63, 95% CI 0.70 to 3.79, I^2^ = 0%, tau2 = 0; RR for stroke 1.12, 95% CI 0.38 to 3.30, I^2^ = 0%, tau2 = 0; see Figure 4). The absolute magnitude of the excess risk of myocardial infarction was relatively low; the risk difference was 0.8% (control group risk = 0.3%) and number needed to harm was 125.

### Thrombosis and thromboembolism

Eight trials (n = 3,747) reported the frequency of any grade thrombotic or thromboembolic events between treatment and control groups; eight trials (n = 6,244) compared the frequency of any grade arterial thrombotic or thromboembolic events; eight trials (n = 5,798) compared any grade venous thrombotic or thromboembolic events and six trials (n = 2,576) compared any or an unspecified grade of pulmonary embolism. The risk of any grade thrombotic or thromboembolic events was significantly higher in VEGFi recipients (RR 1.53, 95% CI 1.07 to 2.20, I^2^ = 43%, tau2 = 0.11) but the absolute increase in risk was relatively low (risk difference 4%; control group risk = 8%) and number needed to harm was 25.

When individual types of thrombotic events were considered separately, the pooled risk among VEGFi recipients was significantly higher for arterial thrombotic events, but not for venous thrombotic events or pulmonary embolism (RR for arterial thrombotic events 1.80, 95% CI 1.24 to 2.59, I^2^ = 0%, tau2 = 0; RR for venous thrombotic events 1.14, 95% CI 0.87 to 1.50, I^2^ = 33%, tau2 = 0.05; RR for pulmonary embolism 1.18, 95% CI 0.51 to 2.73, I^2^ = 48%, tau2 = 0.46; see Figure 4). The absolute increase in the risk of arterial thromboembolism was relatively low (risk difference 1%; control group risk = 2%). In meta-regression, the excess risk of thrombotic event appeared to be greater (p = 0.02) for PTK/ZK (RR 6.52, 95% CI 2.29 to 18.51) than for the other 2 VEGFi agents (bevacizumab and vandetanib, for which the pooled RR was 1.36, 95% CI 1.11 to 1.67, I^2^ = 0%, tau2 = 0). The same panel of potential explanatory variables was considered as in the meta-regression analyses on mortality. However, none significantly modified the association between VEGFi treatment and the risk of myocardial infarction or thrombosis.

### Hypertension and proteinuria

**Figure 5:**
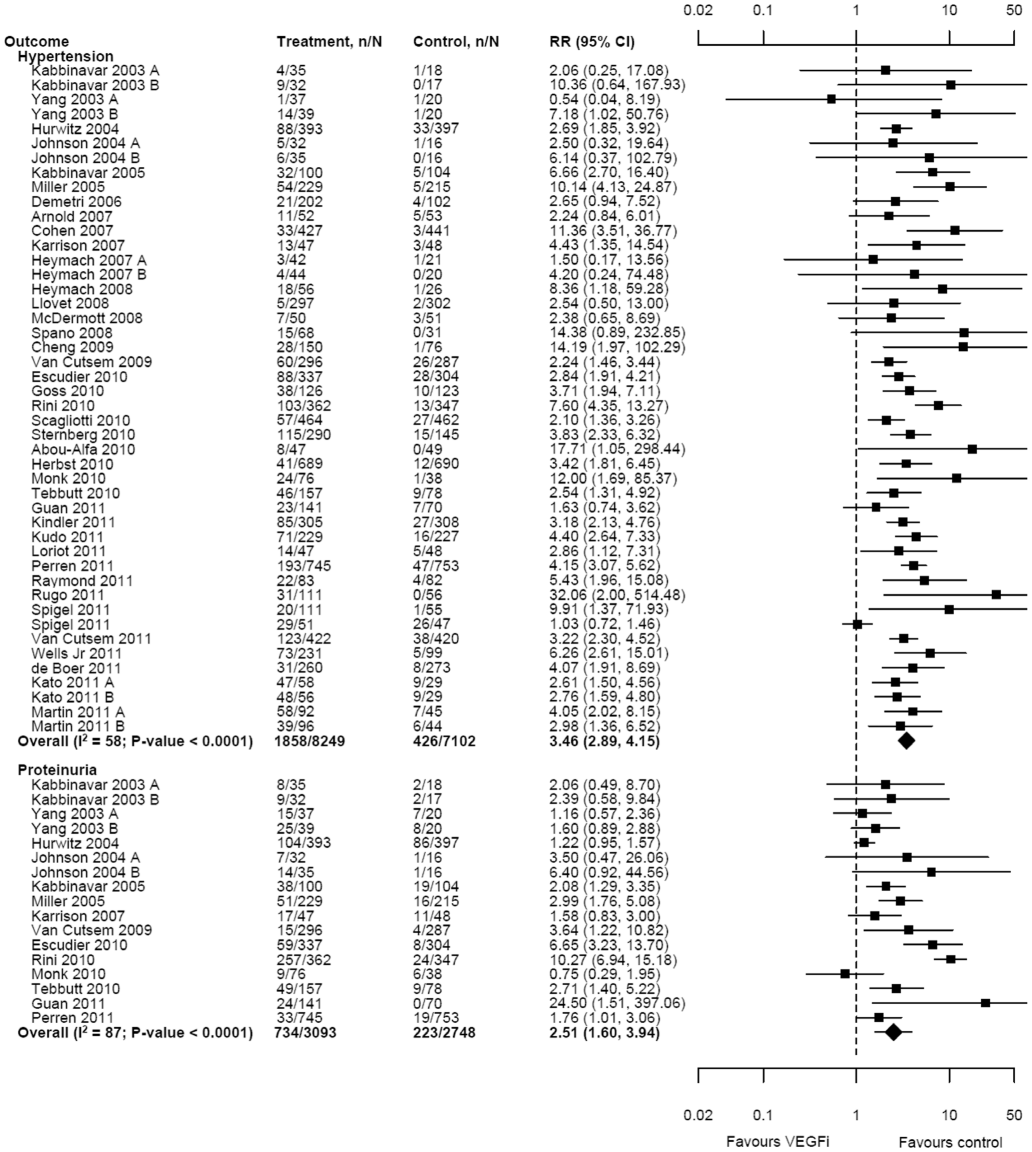
Effect of treatment with VEGFi on hypertension and proteinuria.

Forty trials (n = 15,351) reported the incidence of hypertension. The risk of hypertension was significantly higher among VEGFi recipients (RR 3.46, 95% CI 2.89 to 4.15, I^2^ = 58%, tau2 = 0.16) (Figure 5); this corresponded to an absolute risk difference of 15% (control group risk = 6%) and number needed to harm of 7. Fourteen trials (n = 5, 841) where 13 trials included bevacizumab and 1 trial included pazopanib reported the incidence of proteinuria. The pooled risk of new proteinuria was significantly higher in the VEGFi groups (RR 2.51, 95% CI 1.60 to 3.94, I^2^ = 87%, tau2 = 0.65) (Figure 5); this corresponded to a risk difference of 12% (control group risk = 8%) and number needed to harm of 8. Meta-regression did not identify any of the candidate explanatory variables as significant modifiers of the association between VEGFi treatment and hypertension or proteinuria.

## Discussion

To our knowledge, this is the first meta-analysis to summarize the risk of adverse effects associated with VEGFi treatment in cancer patients. We found that the risks of fatal and nonfatal MI, hypertension, arterial thromboembolism and proteinuria were all higher among VEGFi recipients. The absolute excess risk due to VEGFi treatment varied between the different harms considered, and ranged from relatively low for myocardial infarction (absolute excess risk 0.8%; number needed to harm 125) to relatively high for new proteinuria (absolute excess risk 12%; number needed to harm 8) and hypertension (absolute excess risk 15%; number needed to harm 7). These potential harms must be considered in the context of the demonstrated benefits associated with VEGFi treatment – such as the significantly reduced risk of mortality observed in our review (absolute risk reduction 2%; number needed to treat 50). Since it is possible that timely detection of these adverse events may mitigate their clinical consequences, physicians should consider the need for follow-up measurements of blood pressure, proteinuria and new symptoms of cardiovascular disease – especially in those at higher baseline vascular risk.

Despite our best efforts, we were generally unable to identify treatment- or trial-level characteristics that were associated with especially high or low risk of toxicity. The exception was PTK/ZK treatment, which appeared to be associated with higher excess risk of thrombotic events than the other agents studied (bevacizumab and vandetanib). Of note, PTK/ZK has not been approved by the FDA for the treatment of any cancer.

Hypertension was a common consequence of treatment with VEGFi, with one excess case for approximately every 7 patients treated. The mechanism for VEGFi-induced hypertension is likely multifactorial [8], [19]–[22]; incident hypertension has been hypothesized to identify patients with a good anti-tumor response to VEGFi treatment [23], although we did not evaluate this in our review. According to the findings of a case cross-over study, blood pressure measurement, especially through home monitoring, would facilitate early detection and appropriate management of blood pressure changes in patients receiving VEGFi therapy [24]. Although VEGFi appear to increase the risk of myocardial infarction, we found no convincing evidence that (as a class) they increase the likelihood of heart failure or stroke. Previous authors have speculated that VEGFi might cause cardiotoxicity through their effects on blood pressure, or alternatively by blocking PDGFR signalling [25]. Similarly, we found an association between VEGFi use and the risk of arterial thromboembolic events, but not with the risk of venous thrombosis, which is generally more common. The link between VEGF inhibition and hypercoagulability is plausible, because VEGFi may expose platelets and coagulation factors (such as von Willebrand factor) to subendothelial procoagulant phospholipids – leading to activation of the hemostatic system [19].

We also found that VEGFi treatment substantially increased the risk of new proteinuria – with one excess case for every 8 patients treated. Of 14 trials that reported on incident proteinuria, 13 used bevacizumab, making it uncertain whether the conclusions can be generalized to other agents. VEGFi-induced proteinuria might result from acute hypertension [8], and also from direct effects of VEGF antagonism on the glomerulus. VEGF is an important determinant of normal glomerular function [26], and experimental models show that blocking renal VEGF results in down-regulation of tight junction proteins such as nephrin, with consequent proteinuria [21], [27], [28].

We did not find an increase in the risk of all-cause mortality due to VEGFi treatment, perhaps because increased risk of death due to vascular events is offset by lower risk of death due to cancer. An interaction between chemotherapy co-intervention and total mortality risk (p = 0.007) might be because some participants received chemotherapy to treat or palliate very advanced cancer. Alternatively, VEGFi such as bevacizumab might interact unfavourably with certain chemotherapeutic agents, increasing the risk of adverse events [29].

To our knowledge, this is the first systematic review of randomized trials that examines the adverse events caused by VEGFi in cancer patients. Prior reviews have focused on the risk of bleeding [30], [31] or venous thromboembolic events [32]; others have been limited to studies of specific cancers [10], [30], [33]–[36] or a particular agent [29]. The consistency of our results regardless of the type of cancer or agent studied argues in favour of a more inclusive approach. Our analysis has several important strengths, including the use of a comprehensive search strategy, as a large search yield (72 analyses studying 11 different VEGFi) and rigorous methods including meta-regression. Finally, we included only randomized controlled trial to reduce the risk of bias.

However, our study has some limitations that should be considered. First, the pooled trials were clinically heterogeneous – including variations in cancer type, VEGFi studied, inclusion/exclusion criteria, study risks of bias, and the treatment strategies used. However, metaregression found little evidence that these differences modified the effect of VEGFi on the outcomes of interest. Second, although there was little statistical heterogeneity of effect for the analyses linking VEGFi with the risk of myocardial infarction or arterial thromboembolism, there was statistical heterogeneity in the magnitude of the excess risk of hypertension and proteinuria. Although the statistical heterogeneity makes it difficult to confidently estimate the precise magnitude of the excess risk, it does not threaten our conclusions: 39/40 trials and 13/14 trials showed at least a trend toward excess hypertension and proteinuria respectively among VEGFi recipients. Third, adverse effects were defined and graded differently between various studies – and follow-up time varied from hours to weeks. However, we used the latest follow-up available in all studies to reduce the risk of bias. Fourth, due to resource limitations, we only considered studies published in English. However, since many trials were international (and most cancer trials are published in English), this is unlikely to have affected our conclusions. Also, we did not consider other outcomes such as bleeding [31], [37]–[40] or delayed wound healing complications [41], [42] but this would less likely to change our inferences about mortality or other included outcomes. Finally, although inclusion of only randomized trials likely strengthened the internal validity of our conclusions, it may have reduced generalizability. The risk of cardiac events attributable to VEGFi treatment was larger in observational studies than in randomized trials – perhaps because of the select nature of trial participants. Since the risk of adverse events tends to be higher in “real world” patients, it is likely that our analyses of absolute excess risks have underestimated their true incidence.

In conclusion, VEGFi increase the risk of potentially important adverse effects in people with cancer, including myocardial infarction, arterial thromboembolism, hypertension, and new proteinuria. These harms should be considered in the context of the known benefits of VEGFi for the treatment of cancer.

## Supporting Information

eTable S1 **Search strategies.**
(DOC)

eTable S2 **VEGFi classification.**
(DOC)

eTable S3 **Details of the studies.**
(DOC)

eTable S4 **Study risks of bias assessment.**
(DOC)

eTable S5 **Results of univariable meta-regressions evaluating the effect of individual covariates on the association between VEGFi treatment and mortality, fatal and no-fatal MI, thrombolysis, hypertension and proteinuria.**
(DOC)

eFigure S1 **Publication bias assessment for all-cause mortality.** Funnel plot for all-cause death. No funnel plot asymmetry (bias = −0.34, p = 0.20). Our funnel plot appears symmetric. Therefore, there are unlikely to be missing studies favouring either VEGFi or no VEGFi treatment.
(TIF)

eReference S1 **References of included trials.**
(DOC)

eChecklist S1 **PRISMA 2009 Checklist_Systematic review of VEGFi.**
(DOC)
