# Supplementary material for: Systematic Review of the Risk of Adverse Outcomes Associated with Vascular Endothelial Growth Factor Inhibitors for the Treatment of Cancer
Source: PLoS One. 2014 Jul 2;9(7):e101145. doi: 10.1371/journal.pone.0101145 (PMC4079504; doi:10.1371/journal.pone.0101145)
Supplement: eTable S1 — Search strategies. (DOC) [file pone.0101145.s001.doc]

**eTable S1. Search strategies**

# Literature search strategies

| **Database and Platform** | **Search Strategies** | **Date and Coverage** |
| --- | --- | --- |
| MEDLINE (OVID)  Endnote 1 to 273  Update: 2612-3081  Update 2: 5331-6011 | 1 (Bevacizumab or Avastin or 216974-75-3 or Immunoglobulin G 1 human-mouse monoclonal rhuMAb-VEGF gamma-chain anti-human vascular endothelial growth factor disulfide human-mouse monoclonal rhuMAb-VEGF light chain dimmer or rhuMAb-VEGF or Immunoglobulin G1 anti-human vascular endothelial growth factor human-mouse monoclonal rhuMAb-VEGF gamma1-chain disulfide human-mouse monoclonal rhuMAb-VEGF light chain dimmer or NSC 704865).mp. [mp=title, original title, abstract, name of substance word, subject heading word]  2 (Sunitinib or Sutent or 557795-19-4 or 1H-Pyrrole-3-carboxamide N-2-diethylamino ethyl-5-Z-5-fluoro-1 2-dihydro-2-oxo-3H-indol-3-ylidene methyl-2 4-dimethyl- or SU 11248 or "SU 011248").mp. [mp=title, original title, abstract, name of substance word, subject heading word]  3 (Sorafenib or Nexavar or 284461-73-0 or 4-4-3-4-chloro-3-trifluoromethylphenyl ureido phenoxy pyridine-2-carboxyllic acid methyamide-4-methylbenzenesulfonate or 4-4-4-Chloro-3-trifluoromethyl phenyl amino carbonyl amino phenoxy-N-methyl-2-pyridinecarboxamide or 4-4-3-4-Chloro-3-trifluoromethyl phenyl ureido phenoxy-N sup 2 methylpyridine-2-carboxamide or 2-Pyridinecarboxamide 4-4-4-chloro-3-trifluoromethyl phenyl amino carbonyl amino phenoxy-N-methyl- or BAY 43-9006).mp. [mp=title, original title, abstract, name of substance word, subject heading word]  4 Vatalanib.mp. or PTK/zk or 212141-54-3.mp. or 1-Phtalazinamine N-4-chlorophenyl-4-4-pyridinylmethyl-.mp. or N-4-Chlorophenyl-4-4-pyridinylmethyl-1-phthalazinamine.mp. or N-4-chlorophenyl-4-pyridin-4-ylmethyl phtalazin-1-amine.mp. or 1-Phthalazinamine, N-4-chlorophenyl-4-4-pyridinylmethyl-.mp. or PTK 787.mp. or ZK-222584.mp. or ZK-232934.mp. or CGP-79787$.mp.  5 (Vatalanib Succinate or 212142-18-2 or 1-Phthalazinamine N-4-chlorophenyl-4-4-pyridinylmethyl-butanedioate 1 1 or Butanedioic acid compd with N-4-chlorophenyl-4-4-pyridinylmethyl-1-phthalazinamine 1 1 or CGP 79787D or PTK 787 or ZK 222584).mp. [mp=title, original title, abstract, name of substance word, subject heading word]  6 (Ranibizumab or Lucentis or 347396-82-1 or Immunoglobulin G1 anti-human vascular endothelial growth factor Fab fragment human-mouse monoclonal rhuFAB V2 gamma1 chain disulfide with human-mouse monoclonal rhuFAB V2 light chain or rhuFAb V2).mp. [mp=title, original title, abstract, name of substance word, subject heading word]  7 (Angiostatin$ or 86090-08-6).mp. [mp=title, original title, abstract, name of substance word, subject heading word]  8 (Angiozyme or 220420-78-0 or RNA Gm-sp-Am-sp-Gm-sp-Um-sp-UM-Gm-Cm-2-C-2-propenyl U-G-A-Um-G-Am-Gm-Gm-Cm-Cm-Gm-Am-Am-Am-Gm-Gm-Cm-Cm-G-Am-Am-A-Gm-Um-Cm-Um-Gm-3-3-1-de 6-amino-9H-purin-9-yl dA).mp. [mp=title, original title, abstract, name of substance word, subject heading word]  9 (Axitinib or 319460-85-0 or N-methyl-23-1E-2-pyridin-2-yl ethenyl-1H-indazol-6-yl sulfanyl benzamide or Benzamide N-methyl-2-3-1E-2-2-pyridinyl ethenyl-1H-indazo-6-yl thio- or N Methyl 2 3 2 2 Pyridinyl Ethenyl 1h Indazol 6 Yl Thio Benzamide or "AG 013736").mp. [mp=title, original title, abstract, name of substance word, subject heading word]  10 (Pazopanib or 444731-52-6 or Benzenesulfonamide 5-4-2 3-dimethyl-2H-indazol-6-yl methylamino-2-pyrimidinyl amino-2-methyl- or pazopanib hydrochloride or GW 786034$ or Sb 710468$).mp. [mp=title, original title, abstract, name of substance word, subject heading word]  11 (Acetylsarcosylglycylvalyl Dextro Alloisoleucylthreonylnorvalylisoleucylarginylproline Ethylamide or Abt 510 or 251579-55-2 or L-Prolinamide, N-acetyl-N-methylglycylglycyl-L-valyl-D-alloisoleucyl-L-threonyl-L-norvalyl-L-isoleucyl-L-arginyl-N-ethyl-).mp. [mp=title, original title, abstract, name of substance word, subject heading word]  12 (Shark cartilage extract AE 941 or Neovastat or 305838-77-1 or AE 941).mp. [mp=title, original title, abstract, name of substance word, subject heading word]  13 (Monoclonal Antibody Dc101 or Dc101).mp. [mp=title, original title, abstract, name of substance word, subject heading word]  14 (Pegaptanib Sodium or Macugen or 222716-86-1 or "Eye 001" or Pegaptanib Octasodium or Pegaptanib or Nx 1838 or 5-Ester of 2-deoxy-2-fluoro C-Gm-Gm-A-A- 2-deoxy-2-luoro U-2-deoxy-2-fluoro C-Am-Gm-2-deoxy-2-fluoro U-Gm-Am-Am- 2-deoxy-2-fluoro U-Gm-2-deoxy-2-fluoro C-2-deoxy-2-fluoro U-2-deoxy-2-fluoro U-Am-2-deoxy-2-fluoro U-Am-2-deoxy-2-fluoro C-Am- 2-deoxy-2-fluoro U-2-deoxy-2-fluoro C-2-deoxy-2-fluoroC-Gm-3-3-dT with alpha alpha-1S-1-5-phosphonooxy pentyl carbamoyl pentane-1 5 diyl bis iminocarbonyl bis omega-methoxypoly oxyethane-1 2-diyl octacosasodium salt or RNA 2-deoxy-2-fluoro C-Gm-Gm-A-A- 2-deoxy-2-fluoro U-2-deoxy-2-fluoro C-Am-Gm-2-deoxy-2-fluoro U-Gm-Am-Am-2-deoxy-2-fluoro U-Gm-2-deoxy-2-fluoro C-2-deoxy-2-fluoro U-2-deoxy-2-fluoro U-Am-2-deoxy-2-fluoro U-Am- 2-deoxy-2-fluoro C-Am-2-deoxy-2-fluoro U-2-deoxy-2-fluoroC-2-deoxy-2-fluoro C-Gm-3-3-dT 5-ester with alpha alpha-4 12-dioxo-6-5 phosphonooxy pentyl amino carbonyl-3 13-dioxa-5 11-diaza-1 15 pentadecanediyl bis omega-methoxypoly oxy-1 2-ethanediyl sodium salt).mp. [mp=title, original title, abstract, name of substance word, subject heading word]  15 (Semaxanib or 194413-58-6 or 3 3 5 Dimethyl 1h Pyrrol 2 Yl Methylene 1 3 Dihydro 2h Indol 2 One or 3 2 4 Dimethylpyrrol 5 Yl Methylidenyl Indolin 2 One or Su 5416 or Sugen 5416 or 2H-Indol-2-one 3-3 5-dimethyl-1H-pyrrol-2-yl methylene- 1 3-dihydro- Z- or 3-Z- 3 5-Dimethylpyrrol-2-yl methylene-2-indolinone or -1-3 5-Dimethyl-1H-pyrrol-2-yl meth-Z-ylidene-2-oxo-2 3-dihydroindole or 2H-Indol-2-one 3-3 5-dimethyl-1H-pyrrol-2-yl methylene-1 3-dihydro- 3Z- or 2H-Indol-2-one 3-3 5-dimethyl-1H-pyrrol-2-yl methylene-1 3-dihydro- Z- or -Z- 3 5-Dimethylpyrrol-2-yl methylene -2-indolinone or Semaxinib or 204005-46-9).mp. [mp=title, original title, abstract, name of substance word, subject heading word]  16 (Squalamine or 148717-90-2 or 3beta 5alpha 7alpha 24R-3-3-4-Aminobutyl amino propyl amio cholestane-7 24-diol 24-hydrogen sulfate or 3-3-4-Aminobutyl amino propyl amio cholestane-7 24-diol 24-hydrogen sulfate 3beta 5alpha 7alpha 24R- or 3beta-N-1-N-3-4-Aminobutyl-1 3-diaminopropane-7alpha 24-dihydroxy-5alpha-cholestane 24-sulfate or Cholestane-7 24-diol 3-3-4-aminobutyl amino propyl amio- 24-hydrogen sulfate 3beta 5alpha 7alpha 24R- or Squalamine Trihydrochloride).mp. [mp=title, original title, abstract, name of substance word, subject heading word]  17 (Vandetanib or Zactima or 443913-73-3 or N-4-Bromo-2-fluorophenyl-6-methoxy-7-1-methyl-4-piperidinyl methoxy-4-quinazolinamine or N-4-Bromo-2-fluorophenyl-6-methoxy-7-1-methylpiperidin-4-yl methoxy quinazolin-4-amine or N 4 Bromo 2 Fluorophenyl 6 Methoxy 7 1 Methyl 4 Piperidinylmethoxy 4 Quinazolinamine or N 4 Bromo 2 Fluorophenyl 6 Methoxy 7 1 Methylpiperidin 4 Ylmethoxy Quinazolin 4 Amine or Zd 6474 or Azd 6474).mp. [mp=title, original title, abstract, name of substance word, subject heading word]  18 (5 6 7 13 Tetrahydro 12 3 Hydroxypropyl 9 Isopropoxymethylindeno 2 1 A Pyrrolo 3 4 C Carbazol 5 12h One Dimethylglycine Ester or 402857-58-3 or Cep 7055 or N N Dimethylglycine 3 5 6 7 13 Tetrahydro 9 1 Methylethoxy Methyl 5 Oxo 12h Indeno 2 1 A Pyrrolo 3 4 C Carbazol 12 Yl Propyl Ester or Glycine N N-dimethyl-3-5 6 7 13-tetrahydro-9-1-methylethoxy methyl -5-oxo-12H-indeno 2 1-a pyrrolo 3 4-c carbazol-12-yl propyl ester).mp. [mp=title, original title, abstract, name of substance word, subject heading word]  19 (3 4 Bromo 2 6 Difluorobenzyloxy 5 3 4 1 Pyrrolidinyl Butyl Ureido 4 Isothiazolecarboxamide or 252003-65-9 or Cp 547632 or Cp 547 632 or 4-Isothiazolecarboxamide 3-4-bromo-2 6-difluorophenyl methoxy -5-4-1-pyrrolidinyl butyl amino carbonyl amino-).mp. [mp=title, original title, abstract, name of substance word, subject heading word]  20 (N 4 Bromo 2 Fluorophenyl 6 Methoxy 7 2 1h 1 2 3 Triazol 1 Yl Ethoxy 4 Quinazolinamine or Zd 4190).mp. [mp=title, original title, abstract, name of substance word, subject heading word]  21 (N 3 3 Dimethyl 6 Indolinyl 2 4 Pyridinylmethylamino Nicotinamide or Motesanib or Motesanib diphosphate or 453562-69-1 or Amg 706 or N-3 3-Dimethyl-2 3-dihydro-1H-indol-6-yl-2-pyridin-4ylmethyl amino pyridine-3-carboxamide or 3-Pyridinecarboxamide N-2 3-dihydro-3 3-dimethyl-1H-indol-6-yl-2-4-pyridinylmethyl amino-).mp. [mp=title, original title, abstract, name of substance word, subject heading word]  22 (2 4 Dimethyl 5 2 Oxo 1h Indol 3 Ylmethylene 3 Pyrrolepropionic Acid or 252916-29-3 or 3-2 4-Dimethyl-5-2-oxo-1 2-dihydro-3H-indol-3-ylidene methyl-1H-pyrrol-3-yl propionic acid or 3-2 4-Dimethyl-5-2-oxo-1 2-dihydroindol-3-ylidenemethyl-1H-pyrrol- 3-yl propionic acid or 3-4-2-Carboxyethyl -3 5-dimethylpyrrol-2-methylidenyl -2-indolinone or 5-1 2-Dihydro-2-oxo-3H-indol-3-ylidene methyl-2 4-dimethyl-1H-pyrrole-3-propanoic acid or 1H-Pyrrole-3-propanoic acid 5-1 2-dihydro-2-oxo-3H-indol-3-ylidene methyl-2 4-dimethyl- or Nsc 702827 or Su 6668 or Tsu 68).mp. [mp=title, original title, abstract, name of substance word, subject heading word]  23 (Endostatin$ or 187888-07-9).mp. [mp=title, original title, abstract, name of substance word, subject heading word]  24 (4 4 Fluoro 2 Methyl 5 Indolyloxy 6 Methoxy 7 3 1 Pyrrolidinyl Propoxy Quinazoline or Cediranib or Recentin or Azd 2171 or 288383-20-0 or Quinazoline 4-4-fluoro-2-methyl-1H-indol-5-yl oxy-6-methoxy-7-3-1-pyrrolidinyl propoxy-).mp. [mp=title, original title, abstract, name of substance word, subject heading word]  25 (Fumagillol Chloroacetylcarbamate or 129298-91-5 or Agm 1470 or Agm 470 or Fumigillin or 6 O Chloroacetylcarbamoyl Fumagillol or Chloroacetylcarbamoyl Fumagillol or Tnp 470 or CCRIS 8049 or DRG-0148 or NSC 642492 or O-Chloroacetylcarbamoyl fumagillol or Carbamic acid chloroacetyl-5-methoxy-4-2-methyl-3-3-methyl-2-butenyl oxiranyl-1-oxaspiro 2 5 oct-6-yl ester 3R-3alpha 4alpha 2R 3R 5beta 6beta-).mp. [mp=title, original title, abstract, name of substance word, subject heading word]  26 (GW 2286 or 601517-74-2 or GW 612286 or GW 612286x).mp. [mp=title, original title, abstract, name of substance word, subject heading word]  27 (2 8 Hydroxy 6 Methoxy 1 Oxo 1h 2 Benzopyran 3 Yl Propionic Acid or Isocoumarin NM-3 or 181427-78-1 or NM 3 isocoumarin or NM-3 or 2-8-Hydroxy-6-methoxy-1-oxo-1H-isochromen-3-yl propionic acid or 1H-2-Benzopyran-3-acetic acid 8-hydroxy-6-methoxy-alpha-methyl-1-oxo-or 8-Hydroxy-6-methoxy-alpha-methyl-1-oxo-1H-2-benzopyran-3-acetic acid).mp. [mp=title, original title, abstract, name of substance word, subject heading word]  28 (IM 862 or Glufanide disodium or 237068-57-4 or L-Tryptophan L-alpha-glutamyl- disodium salt or L-tryptophan L-alpha-glutamyl- disodium salt or L-alpha-glutamyl-L-tryptophan disodium salt).mp. [mp=title, original title, abstract, name of substance word, subject heading word]  29 (Lenalidomide or 191732-72-6 or Revimid or Revlimid or 2 6-Piperidinedione 3-4-amino-1 3-dihydro-1-oxo-2H-isoindol-2-yl- or 3-4-Amino-1-oxo-1 3-dihydro-2H-isoindol-2-yl piperidine-2 6-dione or 3-4-Amino-1-oxoisoindolin-2-yl piperidine-2 6-dione or 2 6-Piperidinedione 3-4-amino-1 3-dihydro-1-oxo-2H-isoindol-2-yl- or Cc 5013 or Cdc 501 or 4 Amino 1 3 Dihydro 1 Oxo 2h Isoindol 2 Yl Glutarimide or 3 4 Amino 1 Oxo 1 3 Dihydro 2h Isoindol 2 Yl 2 6 Piperidinedione or Cdc 5013 or "Enmd 0997" or Imid3).mp. [mp=title, original title, abstract, name of substance word, subject heading word]  30 ((Vasculotropin adj2 inhibit$) or (Vascular endothelial growth factor adj2 inhibit$) or (VEGF adj2 inhibitor$) or (Vascular endothelial growth factor receptor tyrosine kinase adj2 inhibit$) or Humanized vascular endothelial growth factor monoclonal antibod$ or (VEGFR tyrosine kinase adj2 inhibit$) or (Anti adj VEGF monoclonal antibod$)).mp. [mp=title, original title, abstract, name of substance word, subject heading word]  31 exp Vascular Endothelial Growth Factors/ai [Antagonists & Inhibitors]  32 or/1-31  33 exp Neoplasms/  34 (cancer$ or neoplas$ or carcino$ or metast$ or sarcom$).mp.  35 33 or 34  36 exp Macular Degeneration/  37 (maculopath$ or macular dystroph$).mp.  38 36 or 37  39 35 or 38  40 limit 39 to "all child (0 to 18 years)"  41 limit 39 to "all adult (19 plus years)"  42 40 not (40 and 41)  43 39 not 42  44 (child$ or pediatric$ or paediatric$).jw.  45 43 not 44  46 randomized controlled trial.pt.  47 controlled clinical trial.pt.  48 meta analysis.pt.  49 exp Randomized Controlled Trials/  50 exp Random Allocation/  51 exp Double-Blind Method/  52 exp Single-Blind Method/  53 exp Meta-Analysis/  54 ((singl$ or doubl$ or tripl$ or trebl$) adj2 (blind$ or mask$)).ti,ab.  55 random$.ti,ab.  56 (systematic adj2 (review$ or overview$)).ti,ab.  57 or/46-56  58 limit 57 to animals  59 limit 57 to (humans and animals)  60 58 not 59  61 57 not 60  62 clinical trial.pt.  63 exp Clinical Trials/  64 (clin$ adj2 trial$).ti,ab.  65 exp Placebos/  66 placebo$.ti,ab.  67 exp Research Design/  68 62 or 63 or 64 or 65 or 66 or 67  69 limit 68 to animals  70 limit 68 to (humans and animals)  71 69 not 70  72 68 not 71  73 comparative study.pt.  74 exp Evaluation Studies/  75 exp Follow-Up Studies/  76 exp Prospective Studies/  77 compar$.ti,ab.  78 or/73-77  79 limit 78 to animals  80 limit 78 to (humans and animals)  81 79 not 80  82 78 not 81  83 61 or 72 or 82  84 32 and 45  85 84 and 83  86 84 not 83  87 exp Cardiovascular Diseases/  88 exp Mortality/  89 mo.fs. or 88  90 87 or 89  91 85 and 90 | 14 Jan 2008  1950 – January Week 1 2008  Update: May 28, 2010  2008-May 2010  Update 2: April 19, 2012 2010-April 2012 |
| EMBASE (OVID)  Endnote 274 – 2358  Update: 3082-5006  Update 2: 6012-7227 | 1 BEVACIZUMAB/ or (Bevacizumab or Avastin or 216974-75-3 or Immunoglobulin G 1 human-mouse monoclonal rhuMAb-VEGF gamma-chain anti-human vascular endothelial growth factor disulfide with human-mouse monoclonal rhuMAb-VEGF light chain dimmer or rhuMAb-VEGF or Immunoglobulin G1 anti-human vascular endothelial growth factor human-mouse monoclonal rhuMAb-VEGF gamma1-chain, disulfide with human-mouse monoclonal rhuMAb-VEGF light chain dimmer or NSC 704865).mp. [mp=title, abstract, subject headings, heading word, drug trade name, original title, device manufacturer, drug manufacturer name]  2 SUNITINIB/ or (Sunitinib or Sutent or 557795-19-4 or 1H-Pyrrole-3-carboxamide N-2-diethylamino ethyl-5-Z-5-fluoro-1 2-dihydro-2-oxo-3H-indol-3-ylidene methyl-2 4-dimethyl- or SU 11248 or "SU 011248").mp. [mp=title, abstract, subject headings, heading word, drug trade name, original title, device manufacturer, drug manufacturer name]  3 SORAFENIB/ or (Sorafenib or Nexavar or 284461-73-0 or 4-4-3-4-chloro-3-trifluoromethylphenyl ureido phenoxy pyridine-2-carboxyllic acid methyamide-4-methylbenzenesulfonate or 4-4-4-Chloro-3-trifluoromethyl phenyl amino carbonyl amino phenoxy-N-methyl-2-pyridinecarboxamide or 4-4-3-4-Chloro-3-trifluoromethyl phenyl ureido phenoxy-N sup 2 methylpyridine-2-carboxamide or 2-Pyridinecarboxamide 4-4-4-chloro-3-trifluoromethyl phenyl amino carbonyl amino phenoxy-N-methyl- or BAY 43-9006).mp. [mp=title, abstract, subject headings, heading word, drug trade name, original title, device manufacturer, drug manufacturer name]  4 VATALANIB/ or (Vatalanib or PTK zk or 212141-54-3 or 1-Phtalazinamine N-4-chlorophenyl-4-4-pyridinylmethyl- or N-4-Chlorophenyl-4-4-pyridinylmethyl-1-phthalazinamine or N-4-chlorophenyl-4-pyridin-4-ylmethyl phtalazin-1-amine or 1-Phthalazinamine N-4-chlorophenyl-4-4-pyridinylmethyl- or PTK 787 or ZK-222584 or ZK-232934 or CGP-79787$).mp. [mp=title, abstract, subject headings, heading word, drug trade name, original title, device manufacturer, drug manufacturer name]  5 (Vatalanib Succinate or 212142-18-2 or 1-Phthalazinamine N-4-chlorophenyl-4-4-pyridinylmethyl-butanedioate 1 1 or Butanedioic acid compd with N-4-chlorophenyl-4-4-pyridinylmethyl-1-phthalazinamine 1 1 or CGP 79787D or PTK 787 or ZK 222584).mp.  6 RANIBIZUMAB/ or (Ranibizumab or Lucentis or 347396-82-1 or Immunoglobulin G1 anti-human vascular endothelial growth factor Fab fragment human-mouse monoclonal rhuFAB V2 gamma1 chain disulfide with human-mouse monoclonal rhuFAB V2 light chain or rhuFAb V2).mp. [mp=title, abstract, subject headings, heading word, drug trade name, original title, device manufacturer, drug manufacturer name]  7 ANGIOSTATIN/ or (Angiostatin$ or 86090-08-6).mp. [mp=title, abstract, subject headings, heading word, drug trade name, original title, device manufacturer, drug manufacturer name]  8 ANGIOZYME/ or (Angiozyme or 220420-78-0 or RNA Gm-sp-Am-sp-Gm-sp-Um-sp-UM-Gm-Cm-2-C-2-propenyl U-G-A-Um-G-Am-Gm-Gm-Cm-Cm-Gm-Am-Am-Am-Gm-Gm-Cm-Cm-G-Am-Am-A-Gm-Um-Cm-Um-Gm-3-3-1-de 6-amino-9H-purin-9-yl dA).mp. [mp=title, abstract, subject headings, heading word, drug trade name, original title, device manufacturer, drug manufacturer name]  9 AXITINIB/ or (Axitinib or 319460-85-0 or N-methyl-23-1E-2-pyridin-2-yl ethenyl-1H-indazol-6-yl sulfanyl benzamide or Benzamide N-methyl-2-3-1E-2-2-pyridinyl ethenyl-1H-indazo-6-yl thio- or N Methyl 2 3 2 2 Pyridinyl Ethenyl 1h Indazol 6 Yl Thio Benzamide or "AG 013736").mp. [mp=title, abstract, subject headings, heading word, drug trade name, original title, device manufacturer, drug manufacturer name]  10 PAZOPANIB/ or (Pazopanib or 444731-52-6 or Benzenesulfonamide, 5-4-2,3-dimethyl-2H-indazol-6-yl methylamino-2-pyrimidinyl amino-2-methyl- or pazopanib hydrochloride or GW 786034$ or Sb 710468$).mp. [mp=title, abstract, subject headings, heading word, drug trade name, original title, device manufacturer, drug manufacturer name]  11 N Acetylsarcosylglycylvalyl Dextro Alloisoleucylthreonylnorvalylisoleucylarginylproline Ethylamide/ or (N Acetylsarcosylglycylvalyl Dextro Alloisoleucylthreonylnorvalylisoleucylarginylproline Ethylamide or Abt 510 or 251579-55-2 or L-Prolinamide, N-acetyl-N-methylglycylglycyl-L-valyl-D-alloisoleucyl-L-threonyl-L-norvalyl-L-isoleucyl-L-arginyl-N-ethyl-).mp. [mp=title, abstract, subject headings, heading word, drug trade name, original title, device manufacturer, drug manufacturer name]  12 Ae 941/ or (Shark cartilage extract AE 941 or Neovastat or 305838-77-1 or AE 941).mp. [mp=title, abstract, subject headings, heading word, drug trade name, original title, device manufacturer, drug manufacturer name]  13 Monoclonal Antibody Dc101/ or (Monoclonal Antibody Dc101 or Dc101).mp. [mp=title, abstract, subject headings, heading word, drug trade name, original title, device manufacturer, drug manufacturer name]  14 PEGAPTANIB/ or (Pegaptanib Sodium or Macugen or 222716-86-1 or "Eye 001" or Pegaptanib Octasodium or Pegaptanib or Nx 1838 or 5'-Ester of 2-deoxy-2-fluoro C-Gm-Gm-A-A- 2-deoxy-2-luoro U-2-deoxy-2-fluoro C-Am-Gm-2-deoxy-2-fluoro U-Gm-Am-Am- 2-deoxy-2-fluoro U-Gm- 2-deoxy-2-fluoro C-2-deoxy-2-fluoro U- 2-deoxy-2-fluoro U-Am-2-deoxy-2-fluoro U-Am-2-deoxy-2-fluoro C-Am- 2-deoxy-2-fluoro U-2-deoxy-2-fluoro C-2-deoxy-2-fluoroC-Gm-3-3-dT with alpha,alpha-1S-1-5-phosphonooxy pentyl carbamoyl pentane-1 5 diyl bis iminocarbonyl bis omega-methoxypoly oxyethane-1 2-diyl octacosasodium salt or RNA 2-deoxy-2-fluoro C-Gm-Gm-A-A- 2-deoxy-2-fluoro U-2-deoxy-2-fluoro C-Am-Gm-2-deoxy-2-fluoro U-Gm-Am-Am-2-deoxy-2-fluoro U-Gm-2-deoxy-2-fluoro C-2-deoxy-2-fluoro U-2-deoxy-2-fluoro U-Am-2-deoxy-2-fluoro U-Am- 2-deoxy-2-fluoro C-Am-2-deoxy-2-fluoro U-2-deoxy-2-fluoroC-2-deoxy-2-fluoro C-Gm-3-3-dT 5-ester with alpha alpha-4 12-dioxo-6-5 phosphonooxy pentyl amino carbonyl-3 13-dioxa-5 11-diaza-1 15 pentadecanediyl bis omega-methoxypoly oxy-1 2-ethanediyl sodium salt).mp. [mp=title, abstract, subject headings, heading word, drug trade name, original title, device manufacturer, drug manufacturer name]  15 SEMAXANIB/ or (Semaxanib or 194413-58-6 or 3 3 5 Dimethyl 1h Pyrrol 2 Yl Methylene 1 3 Dihydro 2h Indol 2 One or 3 2 4 Dimethylpyrrol 5 Yl Methylidenyl Indolin 2 One or Su 5416 or Sugen 5416 or 2H-Indol-2-one 3-3 5-dimethyl-1H-pyrrol-2-yl methylene- 1 3-dihydro-, Z- or 3-Z- 3 5-Dimethylpyrrol-2-yl methylene-2-indolinone or -1-3 5-Dimethyl-1H-pyrrol-2-yl meth-Z-ylidene-2-oxo-2 3-dihydroindole or 2H-Indol-2-one 3-3 5-dimethyl-1H-pyrrol-2-yl methylene-1 3-dihydro- 3Z- or 2H-Indol-2-one 3-3 5-dimethyl-1H-pyrrol-2-yl methylene-1 3-dihydro- Z- or -Z- 3 5-Dimethylpyrrol-2-yl methylene -2-indolinone or Semaxinib or 204005-46-9).mp. [mp=title, abstract, subject headings, heading word, drug trade name, original title, device manufacturer, drug manufacturer name]  16 SQUALAMINE/ or (Squalamine or 148717-90-2 or 3beta 5alpha 7alpha 24R-3-3-4-Aminobutyl amino propyl amio cholestane-7 24-diol 24-hydrogen sulfate or 3-3-4-Aminobutyl amino propyl amio cholestane-7 24-diol 24-hydrogen sulfate 3beta 5alpha 7alpha 24R- or 3beta-N-1-N-3-4-Aminobutyl-1 3-diaminopropane-7alpha 24-dihydroxy-5alpha-cholestane 24-sulfate or Cholestane-7 24-diol 3-3-4-aminobutyl amino propyl amio- 24-hydrogen sulfate 3beta 5alpha 7alpha 24R- or Squalamine Trihydrochloride).mp. [mp=title, abstract, subject headings, heading word, drug trade name, original title, device manufacturer, drug manufacturer name]  17 VANDETANIB/ or (Vandetanib or Zactima or 443913-73-3 or N-4-Bromo-2-fluorophenyl-6-methoxy-7-1-methyl-4-piperidinyl methoxy-4-quinazolinamine or N-4-Bromo-2-fluorophenyl-6-methoxy-7-1-methylpiperidin-4-yl methoxy quinazolin-4-amine or N 4 Bromo 2 Fluorophenyl 6 Methoxy 7 1 Methyl 4 Piperidinylmethoxy 4 Quinazolinamine or N 4 Bromo 2 Fluorophenyl 6 Methoxy 7 1 Methylpiperidin 4 Ylmethoxy Quinazolin 4 Amine or Zd 6474 or Azd 6474).mp. [mp=title, abstract, subject headings, heading word, drug trade name, original title, device manufacturer, drug manufacturer name]  18 "5,6,7,13 Tetrahydro 12 (3 Hydroxypropyl) 9 Isopropoxymethylindeno[2,1 A]Pyrrolo[3,4 C]Carbazol 5(12h) One"/ or (5 6 7 13 Tetrahydro 12 3 Hydroxypropyl 9 Isopropoxymethylindeno 2 1 A Pyrrolo 3 4 C Carbazol 5 12h One Dimethylglycine Ester or 402857-58-3 or Cep 7055 or N N Dimethylglycine 3 5 6 7 13 Tetrahydro 9 1 Methylethoxy Methyl 5 Oxo 12h Indeno 2,1 A Pyrrolo 3 4 C Carbazol 12 Yl Propyl Ester or Glycine N N-dimethyl-3-5 6 7 13-tetrahydro-9-1-methylethoxy methyl -5-oxo-12H-indeno 2 1-a pyrrolo 3 4-c carbazol-12-yl propyl ester).mp. [mp=title, abstract, subject headings, heading word, drug trade name, original title, device manufacturer, drug manufacturer name]  19 "3 (4 Bromo 2,6 Difluorobenzyloxy) 5 [3 [4 (1 Pyrrolidinyl)Butyl]Ureido] 4 Isothiazolecarboxamide"/ or (3 4 Bromo 2 6 Difluorobenzyloxy 5 3 4 1 Pyrrolidinyl Butyl Ureido 4 Isothiazolecarboxamide or 252003-65-9 or Cp 547632 or Cp 547 632 or 4-Isothiazolecarboxamide 3-4-bromo-2 6-difluorophenyl methoxy -5-4-1-pyrrolidinyl butyl amino carbonyl amino-).mp. [mp=title, abstract, subject headings, heading word, drug trade name, original title, device manufacturer, drug manufacturer name]  20 "N (4 Bromo 2 Fluorophenyl) 6 Methoxy 7 [2 (1h 1,2,3 Triazol 1 Yl)Ethoxy] 4 Quinazolinamine"/ or (N 4 Bromo 2 Fluorophenyl 6 Methoxy 7 2 1h 1 2 3 Triazol 1 Yl Ethoxy 4 Quinazolinamine or Zd 4190).mp. [mp=title, abstract, subject headings, heading word, drug trade name, original title, device manufacturer, drug manufacturer name]  21 "N (3,3 Dimethyl 6 Indolinyl) 2 (4 Pyridinylmethylamino)Nicotinamide"/ or (N 3 3 Dimethyl 6 Indolinyl 2 4 Pyridinylmethylamino Nicotinamide or Motesanib or Motesanib diphosphate or 453562-69-1 or Amg 706 or N-3 3-Dimethyl-2 3-dihydro-1H-indol-6-yl-2-pyridin-4ylmethyl amino pyridine-3-carboxamide or 3-Pyridinecarboxamide N-2 3-dihydro-3 3-dimethyl-1H-indol-6-yl-2-4-pyridinylmethyl amino-).mp. [mp=title, abstract, subject headings, heading word, drug trade name, original title, device manufacturer, drug manufacturer name]  22 "2,4 Dimethyl 5 (2 Oxo 1h Indol 3 Ylmethylene) 3 Pyrrolepropionic Acid"/ or (2 4 Dimethyl 5 2 Oxo 1h Indol 3 Ylmethylene 3 Pyrrolepropionic Acid or 252916-29-3 or 3-2 4-Dimethyl-5-2-oxo-1 2-dihydro-3H-indol-3-ylidene methyl-1H-pyrrol-3-yl propionic acid or 3-2 4-Dimethyl-5-2-oxo-1 2-dihydroindol-3-ylidenemethyl-1H-pyrrol- 3-yl propionic acid or 3-4-2-Carboxyethyl -3 5-dimethylpyrrol-2-methylidenyl -2-indolinone or 5-1 2-Dihydro-2-oxo-3H-indol-3-ylidene methyl-2 4-dimethyl-1H-pyrrole-3-propanoic acid or 1H-Pyrrole-3-propanoic acid 5-1 2-dihydro-2-oxo-3H-indol-3-ylidene methyl-2 4-dimethyl- or Nsc 702827 or Su 6668 or Tsu 68).mp. [mp=title, abstract, subject headings, heading word, drug trade name, original title, device manufacturer, drug manufacturer name]  23 ENDOSTATIN/ or (Endostatin$ or 187888-07-9).mp. [mp=title, abstract, subject headings, heading word, drug trade name, original title, device manufacturer, drug manufacturer name]  24 "4 (4 Fluoro 2 Methyl 5 Indolyloxy) 6 Methoxy 7 [3 (1 Pyrrolidinyl)Propoxy]Quinazoline"/ or (4 4 Fluoro 2 Methyl 5 Indolyloxy 6 Methoxy 7 3 1 Pyrrolidinyl Propoxy Quinazoline or Cediranib or Recentin or Azd 2171 or 288383-20-0 or Quinazoline 4-4-fluoro-2-methyl-1H-indol-5-yl oxy-6-methoxy-7-3-1-pyrrolidinyl propoxy-).mp. [mp=title, abstract, subject headings, heading word, drug trade name, original title, device manufacturer, drug manufacturer name]  25 Fumagillol Chloroacetylcarbamate/ or (Fumagillol Chloroacetylcarbamate or 129298-91-5 or Agm 1470 or Agm 470 or Fumigillin or 6 O Chloroacetylcarbamoyl Fumagillol or Chloroacetylcarbamoyl Fumagillol or Tnp 470 or CCRIS 8049 or DRG-0148 or NSC 642492 or O-Chloroacetylcarbamoyl fumagillol or Carbamic acid chloroacetyl-5-methoxy-4-2-methyl-3-3-methyl-2-butenyl oxiranyl-1-oxaspiro 2 5 oct-6-yl ester 3R-3alpha 4alpha 2R 3R 5beta 6beta-).mp. [mp=title, abstract, subject headings, heading word, drug trade name, original title, device manufacturer, drug manufacturer name]  26 GW 2286/ or (GW 2286 or 601517-74-2 or GW 612286 or GW 612286x).mp. [mp=title, abstract, subject headings, heading word, drug trade name, original title, device manufacturer, drug manufacturer name]  27 "2 (8 Hydroxy 6 Methoxy 1 Oxo 1h 2 Benzopyran 3 Yl)Propionic Acid"/ or (2 8 Hydroxy 6 Methoxy 1 Oxo 1h 2 Benzopyran 3 Yl Propionic Acid or Isocoumarin NM-3 or 181427-78-1 or NM 3 isocoumarin or NM-3 or 2-8-Hydroxy-6-methoxy-1-oxo-1H-isochromen-3-yl propionic acid or 1H-2-Benzopyran-3-acetic acid 8-hydroxy-6-methoxy-alpha-methyl-1-oxo-or 8-Hydroxy-6-methoxy-alpha-methyl-1-oxo-1H-2-benzopyran-3-acetic acid).mp. [mp=title, abstract, subject headings, heading word, drug trade name, original title, device manufacturer, drug manufacturer name]  28 Im 862/ or (IM 862 or Glufanide disodium or 237068-57-4 or L-Tryptophan L-alpha-glutamyl- disodium salt or L-tryptophan L-alpha-glutamyl- disodium salt or L-alpha-glutamyl-L-tryptophan disodium salt).mp. [mp=title, abstract, subject headings, heading word, drug trade name, original title, device manufacturer, drug manufacturer name]  29 LENALIDOMIDE/ or (Lenalidomide or 191732-72-6 or Revimid or Revlimid or 2 6-Piperidinedione 3-4-amino-1 3-dihydro-1-oxo-2H-isoindol-2-yl- or 3-4-Amino-1-oxo-1 3-dihydro-2H-isoindol-2-yl piperidine-2 6-dione or 3-4-Amino-1-oxoisoindolin-2-yl piperidine-2 6-dione or 2 6-Piperidinedione 3-4-amino-1 3-dihydro-1-oxo-2H-isoindol-2-yl- or Cc 5013 or Cdc 501 or 4 Amino 1 3 Dihydro 1 Oxo 2h Isoindol 2 Yl Glutarimide or 3 4 Amino 1 Oxo 1 3 Dihydro 2h Isoindol 2 Yl 2 6 Piperidinedione or Cdc 5013 or "Enmd 0997" or Imid3).mp. [mp=title, abstract, subject headings, heading word, drug trade name, original title, device manufacturer, drug manufacturer name]  30 Vasculotropin Inhibitor/ or ((Vasculotropin adj2 inhibit$) or (Vascular endothelial growth factor adj2 inhibit$) or (VEGF adj2 inhibit$) or (Vascular endothelial growth factor receptor tyrosine kinase adj2 inhibit$) or Humanized vascular endothelial growth factor monoclonal antibod$ or (VEGFR tyrosine kinase adj2 inhibit$) or (Anti adj VEGF monoclonal antibod$)).mp. [mp=title, abstract, subject headings, heading word, drug trade name, original title, device manufacturer, drug manufacturer name]  31 or/1-30  32 exp Neoplasm/  33 (cancer$ or neoplas$ or carcino$ or metast$ or sarcom$).mp.  34 32 or 33  35 exp Retina Macula Degeneration/  36 (maculopath$ or (macula$ adj2 dystroph$) or (macula$ adj2 atroph$) or (heredomacula$ adj2 degeneration) or Junius Kuhnt Disease or (macula$ adj2 degeneration)).mp.  37 35 or 36  38 34 or 37  39 31 and 38  40 limit 39 to (embryo <first trimester> or infant <to one year> or child <unspecified age> or preschool child <1 to 6 years> or school child <7 to 12 years> or adolescent <13 to 17 years>)  41 (child$ or pediatric$ or paediatric$).jw,mp.  42 39 and 41  43 40 or 42  44 39 not 43  45 exp Cardiovascular Disease/si [Side Effect]  46 exp MORTALITY/  47 45 or 46  48 44 and 47  49 Randomized Controlled Trial/  50 exp Cohort Analysis/  51 exp Prospective Study/  52 exp Comparative Study/  53 49 or 50 or 51 or 52  54 48 and 53  55 Randomized Controlled Trial/  56 exp RANDOMIZATION/  57 Double Blind Procedure/  58 Single Blind Procedure/  59 Meta Analysis/  60 exp "Systematic Review"/  61 ((singl$ or doubl$ or tripl$ or trebl$) adj2 (blind$ or mask$)).ti,ab.  62 random$.ti,ab.  63 (systematic adj2 (review$ or overview$)).ti,ab.  64 Clinical Trial/  65 (clin$ adj2 trial$).ti,ab.  66 exp PLACEBO/  67 placebo$.ti,ab.  68 exp Comparative Study/  69 exp EVALUATION/  70 exp Follow-Up/  71 exp Prospective Study/  72 compar$.ti,ab.  73 or/55-72  74 exp ANIMAL/  75 Animal Experiment/  76 Animal Model/  77 or/74-76  78 Human/  79 77 and 78  80 77 not 79  81 73 not 80  82 48 and 81  83 82 not 54  84 Clinical Trial/  85 53 or 84  86 85 and 48 | 14 Jan 2008  1988 to 2008 Week 2  Update: May 28, 2010  2008-May 2010  Update: April 19, 2012  2010-April 2012 |
| Cochrane Library (Wiley)  Endnote 2359 to 2370  Update: 5007-5073  Update 2: 7228-7386 | 1 [(Bevacizumab or Avastin or 216974-75-3 or NSC 704865)](http://www3.interscience.wiley.com/cochrane/searchHistory?mode=runquery&qnum=1)  2 [(Sunitinib or Sutent or 557795-19-4 or SU 11248 or SU 011248)](http://www3.interscience.wiley.com/cochrane/searchHistory?mode=runquery&qnum=2)  3 [(Vatalanib or PTK/zk or 212141-54-3 or PTK 787 or ZK-222584 or ZK-232934 or CGP-79787*)](http://www3.interscience.wiley.com/cochrane/searchHistory?mode=runquery&qnum=3)  4 [(Sorafenib or Nexavar or 284461-73-0 or BAY 43-9006)](http://www3.interscience.wiley.com/cochrane/searchHistory?mode=runquery&qnum=4)  5 [(Ranibizumab or Lucentis or 347396-82-1 or rhuFAb V2)](http://www3.interscience.wiley.com/cochrane/searchHistory?mode=runquery&qnum=5)  6 [(Angiostatin* or 86090-08-6)](http://www3.interscience.wiley.com/cochrane/searchHistory?mode=runquery&qnum=6)  7 [(Angiozyme or 220420-78-0 )](http://www3.interscience.wiley.com/cochrane/searchHistory?mode=runquery&qnum=7)  8 [(Axitinib or 319460-85-0 or AG 013736)](http://www3.interscience.wiley.com/cochrane/searchHistory?mode=runquery&qnum=8)  9 [(Pazopanib or 444731-52-6 or GW 786034* or Sb 710468*)](http://www3.interscience.wiley.com/cochrane/searchHistory?mode=runquery&qnum=9)  10 [(Acetylsarcosylglycylvalyl Dextro Alloisoleucylthreonylnorvalylisoleucylarginylproline Ethylamide or Abt 510 or 251579-55-2 )](http://www3.interscience.wiley.com/cochrane/searchHistory?mode=runquery&qnum=10)  11 [(Neovastat or 305838-77-1 or "AE 941")](http://www3.interscience.wiley.com/cochrane/searchHistory?mode=runquery&qnum=11)  12 [(Dc101)](http://www3.interscience.wiley.com/cochrane/searchHistory?mode=runquery&qnum=12)  13 [(Pegaptanib or Macugen or 222716-86-1 or "Eye 001" or "Nx 1838" )](http://www3.interscience.wiley.com/cochrane/searchHistory?mode=runquery&qnum=13)  14 [(Semaxanib or 194413-58-6 or Semaxinib or 204005-46-9)](http://www3.interscience.wiley.com/cochrane/searchHistory?mode=runquery&qnum=14)  15 [(Squalamine or 148717-90-2 )](http://www3.interscience.wiley.com/cochrane/searchHistory?mode=runquery&qnum=15)  16 [(Vandetanib or Zactima or 443913-73-3 or Zd 6474 or Azd 6474)](http://www3.interscience.wiley.com/cochrane/searchHistory?mode=runquery&qnum=16)  17 [(402857-58-3 or Cep 7055 )](http://www3.interscience.wiley.com/cochrane/searchHistory?mode=runquery&qnum=17)  18 [(252003-65-9 or Cp 547 632)](http://www3.interscience.wiley.com/cochrane/searchHistory?mode=runquery&qnum=18)  19 [(Zd 4190)](http://www3.interscience.wiley.com/cochrane/searchHistory?mode=runquery&qnum=19)  20 [(Motesanib or 453562-69-1 or Amg 706 )](http://www3.interscience.wiley.com/cochrane/searchHistory?mode=runquery&qnum=20)  21 [(252916-29-3 or "Nsc 702827" or "Su 6668" or "Tsu 68")](http://www3.interscience.wiley.com/cochrane/searchHistory?mode=runquery&qnum=21)  22 [(Endostatin* or 187888-07-9)](http://www3.interscience.wiley.com/cochrane/searchHistory?mode=runquery&qnum=22)  23 [(Cediranib or Recentin or Azd 2171 or 288383-20-0 )](http://www3.interscience.wiley.com/cochrane/searchHistory?mode=runquery&qnum=23)  24 [(216974-75-3 )](http://www3.interscience.wiley.com/cochrane/searchHistory?mode=runquery&qnum=24)  25 [Fumagillol or 129298-91-5 or "Agm 1470" or "Agm 470" or Fumigillin or "Tnp 470" or "CCRIS 8049" or "DRG-0148" or "NSC 642492"](http://www3.interscience.wiley.com/cochrane/searchHistory?mode=runquery&qnum=25)  26 ["GW 2286" or 601517-74-2 or "GW 612286" or "GW 612286x"](http://www3.interscience.wiley.com/cochrane/searchHistory?mode=runquery&qnum=26)  27 [(181427-78-1 or NM 3 isocoumarin )](http://www3.interscience.wiley.com/cochrane/searchHistory?mode=runquery&qnum=27)  28 ["IM 862" or "Glufanide disodium" or 237068-57-4](http://www3.interscience.wiley.com/cochrane/searchHistory?mode=runquery&qnum=28)  29 [(Lenalidomide or 191732-72-6 or Revimid or Revlimid or "Cc 5013" or "Cdc 501" or "Cdc 5013" or "Enmd 0997" or Imid3)](http://www3.interscience.wiley.com/cochrane/searchHistory?mode=runquery&qnum=29)  30 [(Vasculotropin NEAR/2 inhibit*) or ("Vascular endothelial growth factor" NEAR/2 inhibit*) or (VEGF NEAR/2 inhibit*) or ("Vascular endothelial growth factor receptor tyrosine kinase" NEAR/2 inhibit*) or ("Humanized vascular endothelial growth factor monoclonal" NEXT antibod*) or ("VEGFR tyrosine kinase" NEAR/2 inhibit*) or (Anti NEXT "VEGF monoclonal"NEXT antibod$):ti,ab,kw](http://www3.interscience.wiley.com/cochrane/searchHistory?mode=runquery&qnum=30)  31 [MeSH descriptor Vascular Endothelial Growth Factors explode all trees with qualifier: AI](http://www3.interscience.wiley.com/cochrane/searchHistory?mode=runquery&qnum=31)  32 [(#1 OR #2 OR #3 OR #4 OR #5 OR #6 OR #7 OR #8 OR #9 OR #10 OR #11 OR #12 OR #13 OR #14 OR #15 OR #16 OR #17 OR #18 OR #19 OR #20 OR #21 OR #22 OR #23 OR #24 OR #25 OR #26 OR #27 OR #28 OR #29 OR #30 OR #31)](http://www3.interscience.wiley.com/cochrane/searchHistory?mode=runquery&qnum=32)  33 [MeSH descriptor Neoplasms explode all trees](http://www3.interscience.wiley.com/cochrane/searchHistory?mode=runquery&qnum=33)  34 [MeSH descriptor Macular Degeneration explode all trees](http://www3.interscience.wiley.com/cochrane/searchHistory?mode=runquery&qnum=34)  35 [**(**#33 OR #34)](http://www3.interscience.wiley.com/cochrane/searchHistory?mode=runquery&qnum=35)  36 [MeSH descriptor Cardiovascular Diseases explode all trees](http://www3.interscience.wiley.com/cochrane/searchHistory?mode=runquery&qnum=36)  37 [MeSH descriptor Mortality explode all trees](http://www3.interscience.wiley.com/cochrane/searchHistory?mode=runquery&qnum=37)  38 [(#36 OR #37)](http://www3.interscience.wiley.com/cochrane/searchHistory?mode=runquery&qnum=38)  39 [(#32 AND #35 AND #38)](http://www3.interscience.wiley.com/cochrane/searchHistory?mode=runquery&qnum=39) | 14 Jan 2008  Update: May 28, 2010  Update 2: April 19, 2012 |
|  |
| Scopus  Endnote 2371 – 2440  Update: 5074-5238  Update 2: 7387-7706 | (TITLE-ABS-KEY(rct OR randomize$ OR cohort$ OR double-blind*)) AND (((TITLE-ABS-KEY(cardiovascular OR cardiac OR "myocardial infarction" OR cva OR stroke)) OR (TITLE-ABS-KEY(mortalit* OR death$ OR survival))) AND ((((TITLE-ABS-KEY((vasculotropin W/2 inhibit*) OR ("Vascular endothelial growth factor" W/2 inhibit*) OR (vegf W/2 inhibit*) OR ("Vascular endothelial growth factor receptor tyrosine kinase" W/2 inhibit*)) OR TITLE-ABS-KEY(("Humanized vascular endothelial growth factor monoclonal" W/0 antibod*) OR ("VEGFR tyrosine kinase" W/2 inhibit*) OR (anti W/2 ("VEGF monoclonal" W/0 antibod*)))) AND SUBJAREA(mult OR agri OR bioc OR immu OR neur OR phar OR mult OR medi OR nurs OR vete OR dent OR heal)) OR (((TITLE-ABS-KEY(bevacizumab OR avastin OR "NSC 704865") OR CASREGNUMBER(216974-75-3))) OR ((TITLE-ABS-KEY(semaxanib OR "Su 5416" OR "Sugen 5416" OR semaxinib) OR CASREGNUMBER("194413-58-6" OR "204005-46-9")) AND SUBJAREA(mult OR agri OR bioc OR immu OR neur OR phar OR mult OR medi OR nurs OR vete OR dent OR heal)) OR ((TITLE-ABS-KEY(pegaptanib OR macugen OR "Eye 001" OR pegaptanib OR "Nx 1838") OR CASREGNUMBER(222716-86-1)) AND SUBJAREA(mult OR agri OR bioc OR immu OR neur OR phar OR mult OR medi OR nurs OR vete OR dent OR heal)) OR (TITLE-ABS-KEY(dc101) AND SUBJAREA(mult OR agri OR bioc OR immu OR neur OR phar OR mult OR medi OR nurs OR vete OR dent OR heal)) OR ((TITLE-ABS-KEY("Ae 941" OR "AE 941" OR neovastat) OR CASREGNUMBER(305838-77-1)) AND SUBJAREA(mult OR agri OR bioc OR immu OR neur OR phar OR mult OR medi OR nurs OR vete OR dent OR heal)) OR ((TITLE-ABS-KEY(abt 510 OR "N Acetylsarcosylglycylvalyl Dextro Alloisoleucylthreonylnorvalylisoleucylarginylproline Ethylamide") OR CASREGNUMBER(251579-55-2)) AND SUBJAREA(mult OR agri OR bioc OR immu OR neur OR phar OR mult OR medi OR nurs OR vete OR dent OR heal)) OR ((TITLE-ABS-KEY(pazopanib OR "GW 786034" OR "Sb 710468") OR CASREGNUMBER(444731-52-6)) AND SUBJAREA(mult OR agri OR bioc OR immu OR neur OR phar OR mult OR medi OR nurs OR vete OR dent OR heal)) OR ((TITLE-ABS-KEY(axitinib OR "AG 013736") OR CASREGNUMBER(319460-85-0)) AND SUBJAREA(mult OR agri OR bioc OR immu OR neur OR phar OR mult OR medi OR nurs OR vete OR dent OR heal))) OR (((TITLE-ABS-KEY(angiozyme) OR CASREGNUMBER(220420-78-0)) AND SUBJAREA(mult OR agri OR bioc OR immu OR neur OR phar OR mult OR medi OR nurs OR vete OR dent OR heal)) OR ((TITLE-ABS-KEY(angiostatin$) OR CASREGNUMBER(86090-08-6)) AND SUBJAREA(mult OR agri OR bioc OR immu OR neur OR phar OR mult OR medi OR nurs OR vete OR dent OR heal)) OR ((TITLE-ABS-KEY(ranibizumab OR lucentis OR "rhuFAb V2") OR CASREGNUMBER(347396-82-1)) AND SUBJAREA(mult OR agri OR bioc OR immu OR neur OR phar OR mult OR medi OR nurs OR vete OR dent OR heal)) OR ((TITLE-ABS-KEY("Vatalanib Succinate" OR "CGP 79787D" OR "PTK 787" OR "ZK 222584") OR CASREGNUMBER(212142-18-2)) AND SUBJAREA(mult OR agri OR bioc OR immu OR neur OR phar OR mult OR medi OR nurs OR vete OR dent OR heal)) OR ((TITLE-ABS-KEY(sorafenib OR nexavar OR "BAY 43-9006") OR CASREGNUMBER(284461-73-0)) AND SUBJAREA(mult OR agri OR bioc OR immu OR neur OR phar OR mult OR medi OR nurs OR vete OR dent OR heal)) OR ((TITLE-ABS-KEY(vatalanib OR "PTK zk" OR "PTK 787" OR zk-222584 OR zk-232934 OR cgp-79787$) OR CASREGNUMBER(212141-54-3)) AND SUBJAREA(mult OR agri OR bioc OR immu OR neur OR phar OR mult OR medi OR nurs OR vete OR dent OR heal)) OR ((TITLE-ABS-KEY(sunitinib OR sutent OR "SU 11248" OR "SU 011248") OR CASREGNUMBER(557795-19-4)) AND SUBJAREA(mult OR agri OR bioc OR immu OR neur OR phar OR mult OR medi OR nurs OR vete OR dent OR heal))) OR (((TITLE-ABS-KEY(squalamine) OR CASREGNUMBER(148717-90-2)) AND SUBJAREA(mult OR agri OR bioc OR immu OR neur OR phar OR mult OR medi OR nurs OR vete OR dent OR heal)) OR ((TITLE-ABS-KEY(vandetanib OR zactima OR "Zd 6474" OR "Azd 6474") OR CASREGNUMBER(443913-73-3)) AND SUBJAREA(mult OR agri OR bioc OR immu OR neur OR phar OR mult OR medi OR nurs OR vete OR dent OR heal)) OR ((TITLE-ABS-KEY("5,6,7,13 Tetrahydro 12 (3 Hydroxypropyl) 9 Isopropoxymethylindeno[2,1 A]Pyrrolo[3,4 C]Carbazol 5(12h) One" OR "Cep 7055") OR CASREGNUMBER(402857-58-3)) AND SUBJAREA(mult OR agri OR bioc OR immu OR neur OR phar OR mult OR medi OR nurs OR vete OR dent OR heal)) OR ((TITLE-ABS-KEY("3 (4 Bromo 2,6 Difluorobenzyloxy) 5 [3 [4 (1 Pyrrolidinyl)Butyl]Ureido] 4 Isothiazolecarboxamide" OR "Cp 547632" OR "Cp 547 632") OR CASREGNUMBER(2003-65-9)) AND SUBJAREA(mult OR agri OR bioc OR immu OR neur OR phar OR mult OR medi OR nurs OR vete OR dent OR heal)) OR (TITLE-ABS-KEY("N (4 Bromo 2 Fluorophenyl) 6 Methoxy 7 [2 (1h 1,2,3 Triazol 1 Yl)Ethoxy] 4 Quinazolinamine" OR "Zd 4190") AND SUBJAREA(mult OR agri OR bioc OR immu OR neur OR phar OR mult OR medi OR nurs OR vete OR dent OR heal)) OR ((TITLE-ABS-KEY("N (3,3 Dimethyl 6 Indolinyl) 2 (4 Pyridinylmethylamino)Nicotinamide" OR motesanib OR "Amg 706") OR CASREGNUMBER(453562-69-1)) AND SUBJAREA(mult OR agri OR bioc OR immu OR neur OR phar OR mult OR medi OR nurs OR vete OR dent OR heal)) OR ((TITLE-ABS-KEY("2,4 Dimethyl 5 (2 Oxo 1h Indol 3 Ylmethylene) 3 Pyrrolepropionic Acid" OR "Nsc 702827" OR "Su 6668" OR "Tsu 68") OR CASREGNUMBER(252916-29-3)) AND SUBJAREA(mult OR agri OR bioc OR immu OR neur OR phar OR mult OR medi OR nurs OR vete OR dent OR heal))) OR (((TITLE-ABS-KEY(endostatin$) OR CASREGNUMBER(187888-07-9)) AND SUBJAREA(mult OR agri OR bioc OR immu OR neur OR phar OR mult OR medi OR nurs OR vete OR dent OR heal)) OR ((TITLE-ABS-KEY("4 (4 Fluoro 2 Methyl 5 Indolyloxy) 6 Methoxy 7 [3 (1 Pyrrolidinyl)Propoxy]Quinazoline" OR cediranib OR recentin OR "Azd 2171") OR CASREGNUMBER(288383-20-0)) AND SUBJAREA(mult OR agri OR bioc OR immu OR neur OR phar OR mult OR medi OR nurs OR vete OR dent OR heal)) OR ((TITLE-ABS-KEY("Fumagillol Chloroacetylcarbamate" OR "Agm 1470" OR "Agm 470" OR fumigillin OR "Chloroacetylcarbamoyl Fumagillol" OR "Tnp 470" OR "CCRIS 8049" OR drg-0148 OR "NSC 642492") OR CASREGNUMBER(129298-91-5)) AND SUBJAREA(mult OR agri OR bioc OR immu OR neur OR phar OR mult OR medi OR nurs OR vete OR dent OR heal)) OR ((TITLE-ABS-KEY("GW 2286" OR "GW 612286" OR "GW 612286x") OR CASREGNUMBER(601517-74-2)) AND SUBJAREA(mult OR agri OR bioc OR immu OR neur OR phar OR mult OR medi OR nurs OR vete OR dent OR heal)) OR ((TITLE-ABS-KEY("2 (8 Hydroxy 6 Methoxy 1 Oxo 1h 2 Benzopyran 3 Yl)Propionic Acid" OR "NM-3") OR CASREGNUMBER(181427-78-1)) AND SUBJAREA(mult OR agri OR bioc OR immu OR neur OR phar OR mult OR medi OR nurs OR vete OR dent OR heal)) OR ((TITLE-ABS-KEY("IM 862" OR "Glufanide disodium") OR CASREGNUMBER(237068-57-4)) AND SUBJAREA(mult OR agri OR bioc OR immu OR neur OR phar OR mult OR medi OR nurs OR vete OR dent OR heal)) OR ((TITLE-ABS-KEY(lenalidomide OR revimid OR revlimid OR "Cc 5013" OR "Cdc 501" OR "Cdc 5013" OR "Enmd 0997" OR imid3) OR CASREGNUMBER(191732-72-6)) AND SUBJAREA(mult OR agri OR bioc OR immu OR neur OR phar OR mult OR medi OR nurs OR vete OR dent OR heal)))) AND (TITLE-ABS-KEY(cancer$ OR metast* OR carcinoma$ OR tumor$ OR neoplas* OR sarcoma$ OR (macula$ W/2 degeneration)) AND SUBJAREA(mult OR agri OR bioc OR immu OR neur OR phar OR mult OR medi OR nurs OR vete OR dent OR heal)))) AND (LIMIT-TO(DOCTYPE, "ar") OR LIMIT-TO(DOCTYPE, "re") OR LIMIT-TO(DOCTYPE, "cp")) AND (LIMIT-TO(SUBJAREA, "MEDI") OR LIMIT-TO(SUBJAREA, "PHAR")) | 14 Jan 2007  Update: May 28, 2010  Update: April 19, 2012 |
| BIOSIS Previews  Endnote 2441 to 2487  Update: 5239-5291  Update 2: 7707-7863 | 1 TS=(Bevacizumab OR Avastin OR NSC 704865 OR Sunitinib OR Sutent OR SU 11248 OR SU 011248 OR Vatalanib OR PTK/zk OR PTK 787 OR ZK-222584 OR ZK-232934 OR CGP-79787* OR Sorafenib OR Nexavar OR BAY 43-9006 OR Ranibizumab OR Lucentis OR rhuFAb V2 OR Angiostatin* OR Angiozyme OR Axitinib OR AG 013736 OR Pazopanib OR GW 786034* or Sb 710468* OR "Acetylsarcosylglycylvalyl Dextro Alloisoleucylthreonylnorvalylisoleucylarginylproline Ethylamide" OR Abt 510 OR Neovastat OR "AE 941" OR Dc101 OR Pegaptanib OR Macugen OR "Eye 001" OR "Nx 1838") DocType=All document types; LitType=All literature types; Language=All languages; Taxa Notes=All Taxa Notes; Database=BIOSIS Previews; Timespan=1969-2008  2 TS=(Semaxanib OR Semaxinib OR Squalamine OR Cp 547 632 OR Zd 4190 OR Motesanib OR Amg 706 OR Vandetanib OR Zactima OR Zd 6474 OR Azd 6474 OR "Nsc 702827" OR "Su 6668" OR "Tsu 68" OR Endostatin* OR Cediranib OR Recentin OR Azd 2171 OR "Agm 1470" OR "Agm 470" OR Fumigillin OR Fumagillol OR "Tnp 470" OR "CCRIS 8049" or "DRG 0148" or "NSC 642492" OR "GW 2286" OR "GW 612286" OR "GW 612286x" OR NM 3 isocoumarin OR "IM 862" OR "Glufanide disodium" OR Lenalidomide OR Revimid OR Revlimid OR "Cc 5013" OR "Cdc 501" OR "Cdc 5013" OR "Enmd 0997" OR Imid3) DocType=All document types; LitType=All literature types; Language=All languages; Taxa Notes=All Taxa Notes; Database=BIOSIS Previews; Timespan=1969-2008  3 CA=(216974-75-3 OR 557795-19-4 OR 212141-54-3 OR 212141-54-3 OR 284461-73-0 OR 347396-82-1 OR 220420-78-0 OR 19460-85-0 OR 444731-52-6 OR 251579-55-2 OR 05838-77-1 OR 222716-86-1 OR 194413-58-6 OR 204005-46-9 OR 148717-90-2 OR 252003-65-9 OR 453562-69-1 OR 443913-73-3 OR 252916-29-3 OR 187888-07-9 OR 288383-20-0 OR 216974-75-3 OR 129298-91-5 OR 601517-74-2 OR 181427-78-1 OR 237068-57-4 OR 191732-72-6) DocType=All document types; LitType=All literature types; Language=All languages; Taxa Notes=All Taxa Notes; Database=BIOSIS Previews; Timespan=1969-2008  4 TS=(("Vascular endothelial growth factor" or VEGF) AND inhibit*) DocType=All document types; LitType=All literature types; Language=All languages; Taxa Notes=All Taxa Notes; Database=BIOSIS Previews; Timespan=1969-2008  5 #1 OR #2 OR #3 OR #4 DocType=All document types; LitType=All literature types; Language=All languages; Taxa Notes=All Taxa Notes; Database=BIOSIS Previews; Timespan=1969-2008  6 #1 OR #2 OR #3 OR #4 DocType=Meeting OR Meeting Paper; LitType=All literature types; Language=English; Taxa Notes=Humans; Database=BIOSIS Previews; Timespan=1969-2008  7 CC=(Neoplasms - Therapeutic agents "and" therapy) OR TS=(Macula* Degeneration OR Cancer* OR Carcinoma* OR Tumor* OR Sarcoma* OR Metast*)DocType=Meeting OR Meeting Paper; LitType=All literature types; Language=English; Taxa Notes=Humans; Database=BIOSIS Previews; Timespan=1969-2008  8 #6 AND #7 DocType=Meeting OR Meeting Paper; LitType=All literature types; Language=English; Taxa Notes=Humans; Database=BIOSIS Previews; Timespan=1969-2008  9 TS=(cardiovascular OR cardiac OR "myocardial infarction" OR CVA OR stroke) OR TS=(mortalit* OR death*OR survival) DocType=All document types; LitType=All literature types; Language=All languages; Taxa Notes=All Taxa Notes; Database=BIOSIS Previews; Timespan=1969-2008  10 #9 AND #8 DocType=All document types; LitType=All literature types; Language=All languages; Taxa Notes=All Taxa Notes; Database=BIOSIS Previews; Timespan=1969-2008  11 TS=(RCT OR randomize$ OR cohort$ OR double-blind*)  DocType=All document types; LitType=All literature types; Language=All languages; Taxa Notes=All Taxa Notes; Database=BIOSIS Previews; Timespan=1969-2008  12 #11 AND #10 DocType=All document types; LitType=All literature types; Language=All languages; Taxa Notes=All Taxa Notes; Database=BIOSIS Previews; Timespan=1969-2008 | 14 Jan 2008  Update: May 28, 2010  Update 2: April 19, 2012 |
| ProQuest Dissertations and Theses  EndNote 2488 – 2557  Update: 5292-5318  Update 2: 7864-7894 | Advanced Search  (VEGF W/7 (Anti OR Inhibit*) ) OR (("Vascular Endothelial Growth" W/1 Factor*) W/7 (Anti OR Inhibit*) ) (in Citation and Abstract)  AND  Cancer* OR Metast* OR Tumor* OR Sarcoma* OR Carcinoma* OR Neoplas* OR (Macula* W/2 Degeneration) (in Citation and Abstract) | 15 Jan 2008  1980 – present  Update: 2008-2010  Update 2: 2010-2012 |
