# Supplementary material for: Systematic Review of the Risk of Adverse Outcomes Associated with Vascular Endothelial Growth Factor Inhibitors for the Treatment of Cancer
Source: PLoS One. 2014 Jul 2;9(7):e101145. doi: 10.1371/journal.pone.0101145 (PMC4079504; doi:10.1371/journal.pone.0101145)
Supplement: eTable S2 — VEGFi classification. (DOC) [file pone.0101145.s002.doc]

**eTable S2. VEGFi classification**

| **Generic** | **Brand** | **Experimental** | **Type/MoA** | **Class** |
| --- | --- | --- | --- | --- |
| aflibercept |  |  | recombinant fusion protein (decoy receptor) | VEGF inhibitor |
| bevacizumab | Avastin |  | humanized monoclonal antibody, binds to VEGF | VEGF inhibitor |
| ranibizumab | Lucentis |  | humanized monoclonal antibody fragment, binds to VEGF | VEGF inhibitor |
|  |  | PTC299 | post-transcriptional VEGF modulator | VEGF inhibitor |
| afatinib |  | BIBW 2992 | irreversible HER/2 + VEGFR inhibitor | VEGF Receptor inhibitor, typical |
| apatinib |  | YN968D1 | VEGFR inhibitor | VEGF Receptor inhibitor, typical |
| axitinib | Inlyta | AG-013736 | multikinase inhibitor | VEGF Receptor inhibitor, typical |
| brivanib |  | BMS-582664 | VEGFR and FGBR inhibitor | VEGF Receptor inhibitor, typical |
| cediranib | Recentin | AZD2171 | VEGFR-2 inhibitor | VEGF Receptor inhibitor, typical |
| dovitinib |  | TKI258 | FGFR and VEGR inhibitor | VEGF Receptor inhibitor, typical |
| icrucumab |  | IMC-18F1 | fully human IgG1 monoclonal antibody, binds to VEGFR1 | VEGF Receptor inhibitor, typical |
| pegaptanib | Macugen |  | PEG-bound oligonucleotides, binds to VEGFr | VEGF Receptor inhibitor, typical |
| ramucirumab |  | IMC-1121B | fully human monoclonal Ig1, binds to VEGFR2 | VEGF Receptor inhibitor, typical |
| sunitinib | Sutent |  | VEGFR kinase inhibitor | VEGF Receptor inhibitor, typical |
| tivozanib |  | AV-951 | pan VEGFR inhibitor | VEGF Receptor inhibitor, typical |
|  |  | BMS-844203 | VEGFR antagonist: adnectin, binds to extracellular domain of VEGFR | VEGF Receptor inhibitor, typical |
| famitinib |  |  | multikinase inhibitor | VEGF Receptor inhibitor, atypical |
| foretinib |  |  | c-met and VEGFR inhibitor | VEGF Receptor inhibitor, atypical |
| lenvatinib |  | E7080 | multikinase inhibitor | VEGF Receptor inhibitor, atypical |
| motesanib |  | AMG706 | VEGFR, PDGFR, SCFR | VEGF Receptor inhibitor, atypical |
| oratinib |  | TSU-68 | VEGFR-2 + other kinase | VEGF Receptor inhibitor, atypical |
| pazopanib | Votrient |  | multikinase inhibitor | VEGF Receptor inhibitor, atypical |
| sorafenib | Nexavar |  | multikinase inhibitor | VEGF Receptor inhibitor, atypical |
| vandetanib | Zactima | ZD6474 | VEGFR and EGFR inhibitor | VEGF Receptor inhibitor, atypical |
| vatalanib |  |  | VEGF selective multikinase | VEGF Receptor inhibitor, atypical |
|  | Vargatef | BIBF 1120 | triple kinase inhibitor (EGFR, VEGFR, PDEGFR) | VEGF Receptor inhibitor, atypical |
|  |  | PTK787 | multikinase inhibitor | VEGF Receptor inhibitor, atypical |
|  |  | BMS-690514 | panHER, (VEGFR) inhibitor | VEGF Receptor inhibitor, atypical |
|  |  | CHIR-258 | multikinase inhibitor | VEGF Receptor inhibitor, atypical |
|  |  | XL184 | c-met and VEGFR inhibitor | VEGF Receptor inhibitor, atypical |
|  |  | CVX-241 | Ang2/VEGF fused with a recombinant antibody | VEGF Receptor inhibitor, atypical |
|  |  | SU14813 | multikinase inhibitor | VEGF Receptor inhibitor, atypical |
|  |  | BAY73-4506 | multikinase inhibitor | VEGF Receptor inhibitor, atypical |
|  |  | CEP-11981 | pan-VEGFR/Tie2 tyrosine kinase inhibitor | VEGF Receptor inhibitor, atypical |
|  |  | AEE788 | ErbB and VEGFR inhibitor | VEGF Receptor inhibitor, atypical |
|  |  | JI-101 | inhibits VEGFR2, PDGFRb and EphRB4 | VEGF Receptor inhibitor, atypical |
|  |  | Neovastat | Inhibits VEGFR-2 and MMP 2, 9 and 12 | VEGF Receptor inhibitor, atypical |
|  |  | IM 862 | Inhibits VEGF production and activates natural killer cells. | VEGF Receptor inhibitor, atypical |
|  |  | KH902 | human recombinant vascular endothelial growth factor receptor-Fc fusion protein (peptide body + VEGF antagonist) | VEGF Receptor inhibitor, atypical |
