# Supplementary material for: Systematic Review of the Risk of Adverse Outcomes Associated with Vascular Endothelial Growth Factor Inhibitors for the Treatment of Cancer
Source: PLoS One. 2014 Jul 2;9(7):e101145. doi: 10.1371/journal.pone.0101145 (PMC4079504; doi:10.1371/journal.pone.0101145)
Supplement: eTable S3 — Details of the studies. (DOC) [file pone.0101145.s003.doc]

**eTable S3.** Details of the studies

| **author year country** | **type of tumour stage(%) metastasis Organ>1(%) ECOG >=1(%)** | **Design: sample size treatment (wk) median follow-up(mo) control  incident population** | **VEGF: type initial dose schedule route** | **cointervention on study previous chemo (%) previous radio (%)** | **Demographics: median age male (%)** |
| --- | --- | --- | --- | --- | --- |
| Kabbinavar 2003  USA | metastatic colorectal cancer - 42 41 | 104 max 48 17 # none  - | bevacizumab  5; 10 mg/kg every 2 wks IV | FU/LV 19 14 | - 57 |
| Yang 2003  USA | metastatic RCC - - 22 | 116 - 27 placebo  previously treated | bevacizumab 3; 10 mg/kg every 2 wks IV | none 22 22 | 53 75 |
| Hurwitz 2004 USA,  New Zealand,  Australia | metastatic colorectal cancer - 62 44 | 813 median 40 18 # placebo  previously untreated | bevacizumab 5 mg/kg every 2 wks IV | IFL 26 15 | mean 59 60 |
| Johnson 2004  USA | NSCLC IIIB (15) IV(85) - 50 | 99 max 18 15 # none  mixed | bevacizumab 7.5; 15 mg/kg every 3 wks IV | carboplatin & paclitaxel - 24 | - 61 |
| Kabbinavar 2005 USA, Australia, New Zealand | metastatic colorectal cancer - 66 72 | 209 median 31 15 # placebo  previously untreated | bevacizumab 5mg/kg every 2 wks IV | FU/LV 20 15 | mean 71 53 |
| Miller 2005 USA | metastatic breast cancer - 50 50 | 462 max 105 15 # none  previously treated | bevacizumab 15mg/kg every 3 wks IV | capecitabine 84 - | mean 51 0 |
| Demetri 2006 USA, Canada,  Australia, Italy, Singapore, UK,  Belgium, France, Netherlands | gastrointestinal stromal tumour - - 55 | 312 - max 14 placebo  previously treated | sunitinib 50mg 1/day  oral | none - - | 57 63 |
| Ratain 2006 USA, UK | RCC I(8) II(26) III(26) IV(35) missing(4) 81 46 | 65 - max 500-600 d placebo  mixed | sorafenib 400 mg 2/day oral | none - 31 | 59 72 |
| Sandler 2006 USA | NSCLC IIIB (13) IV(76) recurrent (11)  53 60 | 850 18 19 none  - | bevacizumab 15mg/kg every 3 wks IV | paclitaxel/carboploatin 0 9 | >=65 y (43%) 54 |
| Arnold 2007 Canada | SCLC extensive(57) limited(43) - 71 | 107 max 24 14 placebo  previously treated | vandetanib 300 mg 1/day oral | none 100 66 | 59 54 |
| Cohen 2007 USA | NSCLC IIIB(12) IV(76) recurrent(11) - 60 | 878 18 11 # none  previously untreated | bevacizumab 15 mg/kg every 3 wks IV | carboplatin & paclitaxel 0 - | 63 54 |
| Escudier 2007 France, USA, Poland, Canada | metastatic RCC - 77 47 | 300 - 13 # placebo  previously treated | Neovastat oral 2/day 120ml | none - 25 | 61 73 |
| Escudier 2007 France, USA, UK,  Poland, Germany | metastatic RCC - 86 53 | 903 median 23 7 placebo  previously treated | sorafenib 400mg 2/day oral | none - 26 | 59 73 |
| Giantonio $ 2007  USA,  South Africa | metastatic colorectal cancer - - 50 | 432 - 28 none previously treated | bevacizumab 10 mg/kg every 2 wk IV | FOLFOX4 100 26 | 61 61 |
| Herbst $ 2007 USA | NSCLC - - 54 | 61 max 52 16 placebo  previously treated | bevacizumab 15 mg/kg every 3 wk IV | docetaxel/pemetrexed 100 - | 64 59 |
| Heymach 2007  USA,  Czech Republic,  Hungary | NSCLC IIIB(28) IV(72) - 63 (WHO) | 127 3 11 # placebo  previously treated | vandetanib 100; 300 mg 1/day oral | docetaxel 100 - | 60 58 |
| Karrison 2007 USA | malignant  mesothelioma - - 51 | 108 median 18 15 # placebo  - | bevacizumab 15 mg/kg every 3 wks - | gemcitabine & cisplatin 0 - | 64 79 |
| Mao 2007 USA | prostate cancer - - - | 71 max 24 6 placebo  previously treated | IM 862 5 mg 2/day intranasally | none - 34 | - 100 |
| Miller 2007 USA, Canada | metastatic breast cancer - 45 - | 673 - 43 none  mixed | bevacizumab 10 mg/kg every 2 wks IV | paclitaxel 64 - | 55 0 |
| Heymach $ 2008  USA, Spain, German, India, South Africa | NSCLS IIIB(11), IV(89) - 60 (WHO) | 86 - 16 placebo  previously untreated | vandetanib 300 mg 1/day oral | paclitaxel & carboplatin - - | 60 70 |
| Llovet 2008 Europe, Australia North America, South America | hepatocellular carcinoma - - 46 | 602 median 20 9 # placebo  previously untreated | sorafenib 400 mg 2/day oral | none <1 5 | 66 87 |
| McDermott 2008 USA | melanoma III: 3%; IV M1a: 10%; IV M1b: 35%; IV M1c: 53% 41 & 39 | 101 median 19 12 # placebo  mixed | sorafenib 400 mg  2/day (200 mg each) oral | dacarbazine 0 26 | 58 70 |
| Saltz 2008 A USA, Canada, UK, Australia, Spain, Austria, Taiwan, Switzerland | metastatic colorectal cancer - 59 41 | 700 median 27 28 placebo  previously untreated | bevacizumab 7.5 mg/kg every 3 wks IV | XELOX - - | 61 60 |
| Saltz 2008 B USA, Canada, UK, Australia, Spain, Austria, Taiwan, Switzerland | metastatic colorectal cancer - 58 42 | 700 median 27 28 placebo  previously untreated | bevacizumab 5 mg/kg every 2 wks IV | FOLFOX-4 - - | 60 56 |
| Spano 2008 France, Spain, UK, Canada, USA, Italy | pancreatic cancer - - >=2 (9%) | 103 median 16 6 # none  - | axitinib 5 mg 2/day oral | gemcitabine 9 2 | 64 50 |
| Allegra 2009 USA, Ireland | colon cancer II (node negative): 25; III (1-3 positive nodes): 46; III (+4 positive nodes): 30 - - | 2670 max 54 mean 29 none  - | bevacizumab 5 mg/kg every 2 wks IV | FOLFOX - - | <60 y (58%) 50 |
| Cheng 2009 Taiwan, China, South Korea | hepatocellular carcinoma - 54 74 | 226 - 6 # placebo  previously untreated | sorafenib 400 mg 2/day oral | none - - | 51 86 |
| Hauschild 2009 Germany, USA, France, Canada, Australia, UK, Netherlands | melanoma III: 2; IV M1a: 8; IV M1b: 22; IV M1c: 69 45 & 46 | 270 median 17 11 # placebo  previously treated | sorafenib 400 mg 2/day oral | carboplatin/paclitaxel 65 30 | 56 63 |
| Horti 2009 Hungary, Germany, Brazil, Sweden, South Africa | metastatic prostate cancer - - - | 86 median 18 max 18 placebo  - | vandetanib 100 mg 1/day oral | docetaxel & prednisolone - - | mean 67 100 |
| Van Cutsem 2009 Belgium, France, Netherlands, Canada, Austria, Switzerland | metastatic pancreatic cancer - - - | 607 median 15 7  placebo  - | bevacizumab 5 mg/kg every 2 wks IV | gemcitabine & erlotinib - 3 | 62 59 |
| Abou-Alfa  2010  USA, UK, Canada, Russia, Argentina, China | hepatocellular carcinoma  -  -  >=2 (8%) | 96  17  10 #  placebo  previously untreated | sorafenib  400 mg  2/day  oral | doxorubicin  0  5 | 65  76 |
| Crown  2010  Ireland, France, UK, Poland, USA | breast cancer  -  -  - | 442  -  17 #  none  previously treated | sunitinib 37.5 mg 1/day - | capecitabine  100  - | -  0 |
| Escudier 2010 Europe, Australia, Israel, Singapore, Taiwan | metastatic RCC - - - | 649 median 42 22 placebo  previously untreated | bevacizumab 10 mg/kg every 2 wks IV | interferon alfa-2a - - | 61 70 |
| Goss 2010 Canada, Brazil, Argentina, Romania, Australia, Singapore | NSCLS - 72 75 | 251 median 20 9 placebo  mixed | cediranib 30 mg 1/day oral | carboplatin/paclitaxel 3 - | 59 58 |
| Herbst  2010  USA, China, Germany, Belgium, Japan, Netherlands | NSCLC  IIIB: 14%; IV:86%  -  64 (WHO) | 1391  12  10 #  placebo  previously treated | vandetanib  100 mg  1/d  oral | docetaxel  100  - | 59  70 |
| Kemeny  2010  USA | metastatic colorectal adenocarcinoma  -  -  - | 73  max 12  30  none  mixed | bevacizumab  5 mg/kg  every 2 wks  IV | HUI with irinotecan or oxaliplatin , fluorouracil, leucovorin  86  - | >= 60 y (29%)  52 |
| Kindler  2010  USA | pancreatic cancer  -  -  63 | 602  9  6 #  placebo  previously untreated | bevacizumab  10 mg/kg  every 2 wks  IV | gemcitabine  -  11 | 64  55 |
| Lu  2010  USA, Canada | NSCLC  IIIA:44.3%; IIIB: 55.7%  -  53 | 379  -  44  placebo  previously untreated | Neovastat  120 ml  2/d  oral | paclitaxel & carboplatin, or cisplatin & vinorelbine  -  - | 63  60 |
| Miles 2010  UK, Australia, Canada, South Korea, Europe | breast cancer - >=3 (47%) 39 | 736 31 25 placebo  mixed | bevacizumab 7.5; 15 mg/kg every 3 wks - | docetaxel 66 - | 55 0 |
| Monk $  2010  USA, Peru, Argentina, Spain, France, Thailand | cervical cancer  I:16%; II:34%;III:40%;  IVA:5%; IVB:7%  -  40 | 115  11  -  none  previously treated | pazopanib  800 mg  1/d  oral | lapatinib  100  - | 49  0 |
| Reck 2010  Germany, Czech Republic, Poland, Canada, Russia, Switzerland, UK | NSCLC IIIB without effusion: 8; IIIB with effusion: 8; IV: 78; recurrent: 8 - 60 | 1043 median 17 13 # placebo  previously untreated | bevacizumab 7.5; 15 mg/kg every 3 wks IV | gemcitabine & cisplatin 0 - | 58 64 |
| Rini 2010 USA & Canada | metastatic RCC  - - 38 | 732 median 32 18 # none  previously untreated | bevacizumab 10 mg/kg every 2 wks IV | interferon alfa - 10 | 61 70 |
| Scagliotti 2010  Italy, Germany, Hungary, Poland, Brazil, Chile, USA | NSCLC IIIB: 9; IV: 91 - 59 | 926 median 18 11 # placebo  - | sorafenib 400 mg 2/day oral | carboplatin/paclitaxel 0 - | 62 63 |
| Serve  2010  Germany | acute myeloid leukemia  -  -  - | 197  max 54  -  placebo  - | sorafenib  400 mg  2/day  - | standard induction chemotherapy + consolidation therapy  -  - | -  - |
| Stathopoulos  2010  Greece | colorectal cancer  IV:100%  40  >=2 (27%) (WHO) | 222  max 24  36  none  previously untreated | bevacizumab 7.5 mg/kg every 3 wks - | irinotecan, 5-FU, leucovorin  0  0 | 65  64 |
| Sternberg 2010 Australia, New Zealand, South Korea, Europe, South America | RCC - 54 58 | 435 median 28 max 20 placebo  mixed | pazopanib 800 mg 1/day oral | none - - | 59 70 |
| Tebbutt $  2010  Australia, New Zealand, USA | metastatic colorectal adenocarcinoma  -  -  42 | 235  30  31  none  previously untreated | bevacizumab 7.5 mg/kg every 3 wks - | capecitabine  26  14 | 68  65 |
| Brufsky 2011 USA | metastatic breast cancer - >=3 (45%) 50 | 684 24 15 placebo  previously treated | bevacizumab 15 mg/kg or 10 mg/kg every 3 wks (15mg/kg) or every 2 wks (10 mg/kg) IV | taxane/gemcitabine/ capecitabine /vinorelbine - - | 55 0 |
| Burger  2011  USA, Canada, South Korea, Japan | ovarian cancer  III (<=1cm) 34.1%; III (>1cm) 40.1%; IV: 25.7%  -  50 (GOC) | 1873  max 63 (bevacizumab-throughout) or max 15 (bevacizumab-initiation)  39 #  placebo  previously untreated | bevacizumab (throughout or initiation)  15 mg/kg  every 3 wks  IV | paclitaxel & carboploatin  0  0 | 60  0 |
| Choueiri  2011  USA | urothelial cancer  -  -  48 | 142  -  7  placebo  previously treated | vandetanib  100 mg  1/day  oral | docetaxel  100  23 | >=65 y (50%)  68 |
| de Boer  2011  Belgium, Australia, Mexico, UK, Philippines, South Africa, Italy, Germany, Taiwan | NSCLC  IIIB: 16%; IV:84%  -  59 (WHO) | 534  15  10 #  placebo  previously treated | vandetanib  100 mg  1/day  oral | pemetrexed  98  - | 60  62 |
| Guan  2011  China | metastatic colorectal cancer  -  60  56 | 214  9  17 #  none  previously untreated | bevacizumab 5 mg/kg every 2 wks IV | irinotecan/5-FU  /leucovorin  47  14 | 52  52 |
| Hecht 2011 USA, Germany, Canada, Hungary, Finland, Qatar | metastatic colorectal adenocarcinoma - - 44 (WHO) | 1168 20 21 placebo  previously untreated | PTK/ZK 1250 mg 1/day  oral | FOLFOX 4 - - | 59 62 |
| Herbst  2011  USA | NSCLC  -  -  61 | 636  -  9 #  placebo  previously treated | bevacizumab 15 mg/kg every 3 wks IV | erlotinib  100  - | 65  54 |
| Kato  2011  Japan | colorectal cancer  IV: 100%  48  22 (WHO) | 172  33  19 #  placebo  previously untreated | cediranib  20; 30 mg  1/d  oral | FOLFOX6  -  - | 64  61 |
| Kim  2011  USA, Switzerland | melanoma  IV: 100%  -  31 | 214  18  18  placebo  previously untreated | bevacizumab 15 mg/kg every 3 wks IV | paclitaxel & carboploatin  -  - | 60  69 |
| Kindler  2011  USA, Japan, Netherlands, France, Canada, South Korea, UK, Belgium | pancreatic cancer  -  -  50 | 630  11  6  placebo  - | axitinib  5mg  2/d  oral | gemcitabine  4  - | 62  60 |
| Kudo  2011  Japan, South Korea | hepatocellular carcinoma  -  -  12 | 458  17  max 36  placebo  previously treated | sorafenib  400 mg  2/day  oral | none  100  - | 70  75 |
| Loriot  2011  France | metastatic pancreatic cancer  -  -  - | 95  -  -  placebo  - | vandetanib  300 mg  1/d  oral | bicalutamide  0  - | -  - |
| Martin  2011 A  Spain, France, Hungary, Ireland, Canada, Germany, India, Poland, USA | breast cancer  -  >=3 (49%)  41 | 138  25  max 30  placebo  mixed | motesanib  125 mg  1/d  oral | paclitaxel  66  - | 55  0 |
| Martin  2011 B  Spain, France, Hungary, Ireland, Canada, Germany, India, Poland, USA | breast cancer  -  >=3 (49%)  42 | 144  18  max 30  placebo  mixed | bevacizumab 10 mg/kg every 2 wks IV | paclitaxel  66  - | 54  0 |
| Ohtsu  2011  Japan, South Korea, Europe, Pan-America | gastric cancer  -  -  >=2 (5%) | 774  mean 27  11 #  placebo  previously untreated | bevacizumab 7.5 mg/kg every 3 wks IV | cisplatin & capecitabine/FU  -  - | 59  67 |
| Perren  2011  UK, Germany, Canada, France, Finland, Australia, Norway, Spain, Denmark, Sweden | ovarian cancer  I/IIA 10%; IIB/IIC: 9%; III: 2%;IIIA: 4%; IIIB: 6%;IIIC:57%; IV: 13%  -  54 | 1528  max 54  28  none  - | bevacizumab 7.5 mg/kg every 3 wks - | paclitaxel & carboploatin  -  - | 57  0 |
| Raymond  2011  France, South Korea, UK, Canada, Taiwan, Germany, USA | pancreatic neuroendocrine tumor  -  35  45 | 171  18  max 20  placebo  mixed | sunitinib 37.5 mg 1/day oral | none  69  12 | 56  48 |
| Robert  2011 A  USA, France, UK, Ukraine, Russia | breast cancer  -  >=3 (44%)  - | 615  34  16  placebo  mixed | bevacizumab 15 mg/kg every 3 wks IV | capecitabine  72  64 | 56  0 |
| Robert  2011 B  USA, France, UK, Ukraine, Russia | breast cancer  -  >=3 (45%)  - | 622  34  19  placebo  mixed | bevacizumab 15 mg/kg every 3 wks IV | taxane-based/anthracycline-based  72  64 | 55  0 |
| Rugo  2011  USA, Spain, Canada, Italy, Germany, UK, India, Czech Republic | breast cancer  IV: 76.2%; recurrent: 23.8%  -  >=2 (4%) | 168  23  max 30  placebo  mixed | axitinib  5 mg  2/d  oral | docetaxel  72  - | 55  0 |
| Spigel  2011  USA | SCLC  -  -  63 | 102  20  8  placebo  previously untreated | bevacizumab 15 mg/kg every 3 wks IV | etoposide + cisplatin/carboplatin  0  6 | 62  55 |
| Spigel  2011  USA | NSCLC  -  -  68 | 166  -  7 #  placebo  previously treated | sorafenib  400 mg  2/day  oral | erlotinib  100  - | 65  53 |
| Van Cutsem  2011  Belgium, Italy, UK, Germany, Canada, USA | metastatic colorectal adenocarcinoma  -  -  47 (WHO) | 855 15 12 # placebo  previously treated | PTK/ZK 1250 mg 1/day oral | FOLFOX 4 100 - | 60  62 |
| Wells Jr  2011  USA, Australia, Germany, Italy, France, Poland, UK | medullary thyroid cancer  -  89  36 (WHO) | 331  90  24  placebo  mixed | vandetanib  300 mg  1/d  oral | none  -  - | 52  57 |
| Yang  2011  China | hepatocellular carcinoma  -  -  - | 102  -  10  none  - | sorafenib  400 mg  2/day  oral | cryotherapy  -  - | -  - |
| Bear  2012  USA, Canada | breast cancer  -  -  - | 1206  max 18  max 54  none  previously untreated | bevacizumab 15 mg/kg every 3 wks IV | docetaxel/docetaxel-capecitabine/docetaxel-gemcitabine  -  - | >= 60y (8%)  0 |
| Kelly  2012  USA | metastatic prostate cancer | 1050  Max 104  25 (after study closure)  None  mixed | Bevacizumab  15 mg/kg every 3 wks IV | docetaxel & prednisone  -  - | >= 65y (67%)  100 |
| von Minckwitz  2012  Germany, Switzerland | breast cancer  -  -  - | 1925  max 24  -  none [none] previously untreated | bevacizumab 15 mg/kg every 3 wks IV | docetaxel + epirubicin-cyclophoshamide  -  - | 48  0 |

NSCLC: non-small-cell lung cancer; RCC: renal cell carcinoma; SCLC: small-cell lung cancer; FOLFOX 4: oxaliplatin, leucovorin, fluorouracil; FU/LV:fluorouracil & Leucovorin; IFL: irinotecan, bolus fluorouracil, leucovorin; XELOX: capecitabine & oxaliplatin

#: median survival

& disease sites >3

$ Comparison between another arm and control was not included.

*These are two different trials published in the same year by the same author: Spigel et al in 2011 used bevacizumab for extensive stage small cell lung cancer in one trial and sorafenib for advanced non–small-cell lung cancer in another trial. Likewise, Escudier et al in 2007 used neovastat in metastatic renal cell carcinoma in one trial and sorafenib in advanced clear-cell renal-cell carcinoma in another trial.
