# Supplementary material for: Systematic Review of the Risk of Adverse Outcomes Associated with Vascular Endothelial Growth Factor Inhibitors for the Treatment of Cancer
Source: PLoS One. 2014 Jul 2;9(7):e101145. doi: 10.1371/journal.pone.0101145 (PMC4079504; doi:10.1371/journal.pone.0101145)
Supplement: eTable S4 — Study risks of bias assessment. (DOC) [file pone.0101145.s004.doc]

**eTable S4. Study risks of bias** assessment

| **Author, year** | **Method of randomization described and appropriate** | **Treatment allocation concealed** | **Participants blinded** | **Described as double-blind** | **Description of losses to follow-up (% of participants)** | **Intention-to-treat** | **Interim or preliminary analysis** | **Funding** |
| --- | --- | --- | --- | --- | --- | --- | --- | --- |
| Kabbinavar 2003 | No | Unclear | Unclear | No | No | No | No | Industry |
| Yang 2003 | No | Unclear | Yes | Yes | Yes (1) | Yes | Yes | Government, Industry |
| Hurwitz 2004 | Yes | Unclear | Yes | No | No | Yes | No | Industry |
| Johnson 2004 | No | Unclear | Unclear | No | No | Yes | No | Industry |
| Kabbinavar 2005 | No | Adequate | Yes | No | Partial (11) | Yes | No | Industry |
| Miller 2005 | No | Unclear | Unclear | No | Yes (4) | Yes | No | Industry |
| Demetri 2006 | No | Adequate | Yes | Yes | Yes (3) | Yes | No | Industry, Foundation |
| Ratain 2006 | No | Adequate | Yes | Yes | No | Yes | No | Industry |
| Sandler 2006 | No | Unclear | Unclear | No | Yes (3) | Yes | No | Government, Industry |
| Arnold 2007 | Yes | Adequate | Yes | Yes | Yes (2) | Yes | No | Government, Society, Industry |
| Cohen 2007 | No | Unclear | No | No | No | Yes | No | Government |
| Escudier 2007 | No | Unclear | Yes | No | No | No | No | Industry |
| Escudier 2007 | No | Unclear | Yes | Yes | Yes (8) | Yes | No | Industry |
| Giantonio 2007 | No | Unclear | No | No | Partial (1) | Yes | No | Government, Industry |
| Heymach 2007 | No | Unclear | Yes | Yes | No | Yes | No | Industry, Foundation |
| Karrison 2007 | No | Unclear | Yes | Yes | No | No | Yes | Government, Foundation |
| Mao 2007 | No | Unclear | Yes | Yes | Partial (15) | No | Yes | Government, Foundation |
| Miller 2007 | No | Unclear | Unclear | No | No | No | Yes | n/r |
| Herbst 2007 | No | Unclear | Yes | No | No | Yes | No | Industry |
| Heymach 2008 | No | Unclear | Yes | No | Partial (n/r) | Yes | No | Industry, Foundation, Society |
| Llovet 2008 | Yes | Adequate | Yes | Yes | Partial (9) | Yes | No | Industry |
| McDermott 2008 | Yes | Adequate | Yes | Yes | Yes (0) | Yes | No | Industry |
| Saltz 2008 | No | Adequate | Yes | No | No | Yes | No | Industry |
| Spano 2008 | No | Adequate | No | No | Partial (13) | Yes | No | Industry |
| Allegra 2009 | No | Unclear | Unclear | No | No | No | No | Government, Industry |
| Cheng 2009 | No | Adequate | Yes | Yes | Yes (17) | Yes | No | Industry |
| Hauschild 2009 | Yes | Adequate | Yes | Yes | Yes (6) | Yes | No | Industry |
| Horti 2009 | No | Unclear | Yes | Yes | No | Yes | No | Industry |
| Van Cutsem 2009 | No | Adequate | Yes | No | Yes (1) | Yes | No | Industry |
| Escudier 2010 | Yes | Adequate | Yes | Yes | Yes (4) | Yes | No | Industry |
| Goss 2010 | Yes | Adequate | Yes | Yes | No | Yes | No | Industry, Society |
| Rini 2010 | Yes | Unclear | Unclear | No | Yes (1) | Yes | No | Government |
| Scagliotti 2010 | No | Unclear | Yes | No | Yes (6) | Yes | No | Industry |
| Sternberg 2010 | No | Adequate | Yes | Yes | Yes (5) | Yes | Yes | Industry, Government, Other |
| Abou-Alfa 2010 | No | Adequate | Yes | Yes | Yes (5) | Yes | No | Industry |
| Crown 2010 | No | Unclear | Unclear | No | No | Yes | No | n/r |
| Herbst 2010 | Yes | Adequate | Yes | Yes | Yes (5) | Yes | No | Industry |
| Kemeny 2010 | No | Unclear | Unclear | No | No | Yes | No | Industry |
| Kindler 2010 | No | Unclear | Yes | Yes | Yes (0) | Yes | No | Government |
| Lu 2010 | Yes | Unclear | Yes | Yes | No | Yes | Yes | Government, Industry |
| Miles 2010 | No | Adequate | Yes | Yes | No | Yes | No | Industry |
| Monk 2010 | No | Unclear | No | No | Partial (n/r) | Yes | No | Industry |
| Reck 2010 | No | Unclear | Yes | Yes | No | Yes | No | Industry |
| Serve 2010 | No | Unclear | Yes | Yes | No | No | No | n/r |
| Stathopoulos 2010 | Yes | Unclear | Unclear | No | Yes (0) | Yes | No | n/r |
| Tebbutt 2010 | No | Unclear | No | No | No | Yes | No | Government, Industry |
| Brufsky 2011 | Yes | Adequate | Yes | Yes | Yes (1) | Yes | No | Industry |
| Burger 2011 | Yes | Unclear | Yes | Yes | No | Yes | No | Government, Industry |
| Choueiri 2011 | Yes | Unclear | Yes | Yes | No | Yes | No | Industry |
| de Boer 2011 | Yes | Unclear | Yes | Yes | No | Yes | No | Industry |
| Guan 2011 | No | Adequate | No | No | No | Yes | No | Industry |
| Hecht 2011 | No | Unclear | Yes | Yes | Yes (13) | Yes | No | Industry |
| Herbst 2011 | Yes | Adequate | Yes | Yes | No | Yes | No | Government, Industry |
| Kato 2011 | No | Unclear | Yes | Yes | No | No | No | Industry |
| Kim 2011 | No | Adequate | Yes | Yes | Partial (0) | Yes | No | Industry |
| Kindler 2011 | Yes | Adequate | Yes | Yes | Yes (1) | Yes | No | Industry |
| Kudo 2011 | No | Unclear | Yes | No | Yes (3) | Yes | No | Industry |
| Loriot 2011 | No | Unclear | Yes | Yes | No | No | No | Industry |
| Martin 2011 | Yes | Adequate | Yes | Yes | Yes (9) | Yes | No | Industry |
| Ohtsu 2011 | Yes | Unclear | Yes | Yes | No | Yes | No | Industry |
| Perren 2011 | Yes | Adequate | Unclear | No | Yes (4) | Yes | No | Government, Industry |
| Raymond 2011 | No | Unclear | Yes | Yes | Yes (1) | Yes | Yes | Industry |
| Robert 2011 | No | Adequate | Yes | Yes | Partial (0) | Yes | No | Industry |
| Rugo 2011 | No | Unclear | Yes | Yes | No | Yes | No | Industry |
| Spigel 2011 | No | Unclear | Yes | Yes | No | Yes | No | Industry |
| Spigel 2011 | No | Adequate | Yes | Yes | No | Yes | No | Industry |
| Van Cutsem 2011 | No | Adequate | Yes | Yes | Yes (9) | Yes | No | Industry |
| Wells 2011 | No | Unclear | Yes | Yes | Yes (9) | Yes | No | Industry |
| Yang 2011 | No | Unclear | No | No | No | No | No | n/r |
| Bear 2012 | Yes | Unclear | Unclear | No | No | No | No | Government, Industry |
| Kelly 2012 | Yes | Unclear | Yes | Yes | No | Yes | No | Industry |
| Von Minckwitz 2012 | Yes | Adequate | Unclear | No | No | Yes | No | Industry |
