# Supplementary material for: Systematic Review of the Risk of Adverse Outcomes Associated with Vascular Endothelial Growth Factor Inhibitors for the Treatment of Cancer
Source: PLoS One. 2014 Jul 2;9(7):e101145. doi: 10.1371/journal.pone.0101145 (PMC4079504; doi:10.1371/journal.pone.0101145)
Supplement: eTable S5 — Results of univariable meta-regressions evaluating the effect of individual covariates on the association between VEGFi treatment and mortality, fatal and no-fatal MI, thrombolysis, hypertension and proteinuria. (DOC) [file pone.0101145.s005.doc]

**eTable S5. Results of univariable meta-regressions evaluating the effect of individual covariates on the association between VEGFi treatment and mortality, fatal and no-fatal MI, thrombolysis, hypertension and proteinuria**

| **Outcomes** | **Potential modifiers** | **Trials, n** | **Patients, n** | **Ratio of Relative Risks (95% CI)** | **P value** |
| --- | --- | --- | --- | --- | --- |
| Mortality | Median age | 35 | 20,583 | 0.98 (0.91, 1.05) per decade | 0.59 |
|  | Percentage of male participants | 37 | 21,523 | 1.00 (0.99, 1.01) per 10% increment | 0.50 |
|  | VEGFi type  VEGF inhibitor  VEGF Receptor inhibitor, typical  VEGF Receptor inhibitor, atypical | 37  --  --  -- | 21,523  --  --  -- | --  Reference  0.69 (0.43, 1.10)  1.02 (0.98, 1.07) | --  --  0.11  0.24 |
|  | Median duration of follow-up | 37 | 21,523 | 1.00 (0.99, 1.00) per 3 months increment | 0.50 |
|  | Median duration of treatment | 29 | 16,317 | 0.99 (0.98, 1.00) per 4 weeks increment | 0.08 |
|  | Incident population  Untreated  Treated  Mixed | 33  --  --  -- | 18,435  --  --  -- | --  Reference  1.04 (1.00, 1.09)  0.99 (0.91, 1.06) | --  --  0.08  0.70 |
|  | Cancer type  NSCLC  Breast Cancer  Colorectal cancer  Other | 37  --  --  --  -- | 21,523  --  --  --  -- | --  Reference  0.97 (0.90, 1.05)  1.01 (0.96, 1.06)  0.97 (0.92, 1.02) | --  --  0.42  0.76  0.29 |
|  | Percentage of participants in stage III | 14 | 9,459 | 1.00 (0.98, 1.01) per 10% increment | 0.47 |
|  | Percentage of participants in metastasis Organ>1 | 8 | 4,726 | 0.98 (0.92, 1.04) per 10% increment | 0.41 |
|  | Percentage of participants with prior chemotherapy | 23 | 13,112 | 1.01 (1.00, 1.02) per 10% increment | 0.09 |
|  | Percentage of participants with prior radiotherapy | 15 | 8,290 | 1.00 (0.96, 1.04) per 10% increment | 0.95 |
|  | Percentage of participants in ECOG>=1 | 29 | 17,345 | 1.01 (0.99, 1.04) per 10% increment | 0.26 |
|  | Jadad scale of study quality | 37 | 21,523 | 1.01 (0.99, 1.03) per 1 point increment | 0.25 |
| Myocardial infarction | Median age | 6 | 3,296 | 0.97 (0.08, 11.27) per decade | 0.97 |
|  | Percentage of male participants | 7 | 4,163 | 0.99 (0.73, 1.35) per 10% increment | 0.93 |
|  | VEGFi type  VEGF inhibitor  VEGF Receptor inhibitor, typical  VEGF Receptor inhibitor, atypical | 7  --  --  -- | 4,163  --  --  -- | --  Reference  --  1.87 (0.21, 16.56) | --  --  --  0.50 |
|  | Median duration of follow-up | 7 | 4,163 | 0.88 (0.60, 1.29) per 3 months increment | 0.42 |
|  | Median duration of treatment | 6 | 3,733 | 0.95 (0.41, 2.20) per 4 weeks increment | 0.86 |
|  | Incident population  Untreated  Treated  Mixed | 6  --  --  -- | 3,296  --  --  -- | --  Reference  0.94 (0.09, 10.02)  -- | --  --  0.94  -- |
|  | Cancer type  NSCLC  Breast Cancer  Colorectal cancer  Other | 7  --  --  --  -- | 4,163  --  --  --  -- | --  Reference  --  0.35 (0.01, 78.26)  1.43 (0.01, 157.6) | --  --  --  0.62  0.84 |
|  | Percentage of participants in stage III | 1 | 867 | -- | -- |
|  | Percentage of participants in metastasis Organ>1 | 3 | 1,979 | 1.26 (0.01, 727.0) per 10% increment | 0.72 |
|  | Percentage of participants with prior chemotherapy | 5 | 2,611 | 0.82 (0.50, 1.32) per 10% increment | 0.28 |
|  | Percentage of participants with prior radiotherapy | 5 | 2,505 | 1.07 (0.12, 9.46) per 10% increment | 0.93 |
|  | Percentage of participants in ECOG>=1 | 5 | 3,418 | 0.96 (0.16, 5.86) per 10% increment | 0.95 |
|  | Jadad scale of study quality | 7 | 4,163 | 1.47 (0.35, 6.17) per 1 point increment | 0.52 |
| Any thrombotic event | Median age | 6 | 3,547 | 0.67 (0.13, 3.44) per decade | 0.53 |
|  | Percentage of male participants | 8 | 3,747 | 0.98 (0.81, 1.18) per 10% increment | 0.77 |
|  | VEGFi type  VEGF inhibitor  VEGF Receptor inhibitor, typical  VEGF Receptor inhibitor, atypical | 8  --  --  -- | 3,747  --  --  -- | --  Reference  --  2.62 (0.73, 9.48) | --  --  --  0.12 |
|  | Median duration of follow-up | 8 | 3,747 | 0.81 (0.42, 1.54) per 3 months increment | 0.47 |
|  | Median duration of treatment | 4 | 1,944 | 0.83 (0.43, 1.61) per 4 weeks increment | 0.34 |
|  | Incident population  Untreated  Treated  Mixed | 5  --  --  -- | 2,052  --  --  -- | --  Reference  2.93 (0.17, 49.47)  1.37 (0.05, 35.51) | --  --  0.31  0.78 |
|  | Cancer type  NSCLC  Breast Cancer  Colorectal cancer  Other | 8  --  --  --  -- | 3,747  --  --  --  -- | --  Reference  --  1.12 (0.17, 7.48)  0.87 (0.12, 6.60) | --  --  --  0.89  0.88 |
|  | Percentage of participants in stage III | 2 | 1,596 | 1.04 (0.22, 4.99) per 10% increment | 0.80 |
|  | Percentage of participants in metastasis Organ>1 | 3 | 1,096 | 0.71 (0.21, 2.39) per 10% increment | 0.35 |
|  | Percentage of participants with prior chemotherapy | 6 | 2,151 | 1.14 (0.92, 1.41) per 10% increment | 0.17 |
|  | Percentage of participants with prior radiotherapy | 5 | 1,299 | 0.81 (0.48, 1.39) per 10% increment | 0.37 |
|  | Percentage of participants in ECOG>=1 | 8 | 3,747 | 0.77 (0.49, 1.20) per 10% increment | 0.21 |
|  | Jadad scale of study quality | 8 | 3,747 | 1.23 (0.75, 2.00) per 1 point increment | 0.36 |
| Hypertension | Median age | 37 | 15,055 | 0.85 (0.57, 1.28) per decade | 0.43 |
|  | Percentage of male participants | 39 | 15,256 | 0.95 (0.88, 1.03) per 10% increment | 0.22 |
|  | VEGFi type  VEGF inhibitor  VEGF Receptor inhibitor, typical  VEGF Receptor inhibitor, atypical | 40  --  --  -- | 15,351  --  --  -- | --  Reference  1.05 (0.63, 1.75)  1.13 (0.75, 1.71) | --  --  0.84  0.54 |
|  | Median duration of follow-up | 38 | 15,142 | 1.05 (0.98, 1.12) per 3 months increment | 0.17 |
|  | Median duration of treatment | 29 | 12,114 | 1.03 (0.97, 1.09) per 4 weeks increment | 0.32 |
|  | Incident population  Untreated  Treated  Mixed | 32  --  --  -- | 11,340  --  --  -- | --  Reference  1.25 (0.75, 2.09)  1.30 (0.75, 2.25) | --  --  0.38  0.33 |
|  | Cancer type  NSCLC  Breast Cancer  Colorectal cancer  Other | 40  --  --  --  -- | 15,351  --  --  --  -- | --  Reference  1.39 (0.64, 3.02)  0.77 (0.43, 1.36)  0.91 (0.55, 1.51) | --  --  0.40  0.35  0.71 |
|  | Percentage of participants in stage III | 11 | 5,899 | 1.07 (1.00, 1.14) per 10% increment | 0.08 |
|  | Percentage of participants in metastasis Organ>1 | 11 | 3,328 | 1.05 (0.80, 1.37) per 10% increment | 0.72 |
|  | Percentage of participants with prior chemotherapy | 29 | 10,272 | 1.04 (0.99, 1.10) per 10% increment | 0.14 |
|  | Percentage of participants with prior radiotherapy | 16 | 4,312 | 0.97 (0.73, 1.28) per 10% increment | 0.80 |
|  | Percentage of participants in ECOG>=1 | 33 | 13,556 | 0.99 (0.87, 1.12) per 10% increment | 0.84 |
|  | Jadad scale of study quality | 40 | 15,351 | 0.99 (0.82, 1.19) per 1 point increment | 0.89 |
| Proteinuria | Median age | 12 | 5,640 | 1.31 (0.56, 3.06) per decade | 0.49 |
|  | Percentage of male participants | 14 | 5,841 | 1.07 (0.90, 1.27) per 10% increment | 0.44 |
|  | VEGFi type  VEGF inhibitor  VEGF Receptor inhibitor, typical  VEGF Receptor inhibitor, atypical | 14  --  --  -- | 5,841  --  --  -- | --  Reference  --  0.28 (0.04, 2.00) | --  --  --  0.19 |
|  | Median duration of follow-up | 13 | 5,727 | 0.93 (0.72, 1.20) per 3 months increment | 0.54 |
|  | Median duration of treatment | 9 | 3,582 | 1.05 (0.76, 1.46) per 4 weeks increment | 0.73 |
|  | Incident population  Untreated  Treated  Mixed | 10  --  --  -- | 3,563  --  --  -- | --  Reference  0.39 (0.09, 1.67)  1.25 (0.12, 13.51) | --  --  0.18  0.84 |
|  | Cancer type  NSCLC  Breast Cancer  Colorectal cancer  Other | 14  --  --  --  -- | 5,841  --  --  --  -- | --  Reference  0.63 (0.05, 8.73)  0.48 (0.06, 3.94)  0.51 (0.07, 3.95) | --  --  0.71  0.46  0.49 |
|  | Percentage of participants in stage III | 3 | 1,711 | 0.85 (0.30, 2.39) per 10% increment | 0.58 |
|  | Percentage of participants in metastasis Organ>1 | 5 | 1,751 | 0.83 (0.37, 1.89) per 10% increment | 0.57 |
|  | Percentage of participants with prior chemotherapy | 9 | 2,311 | 1.01 (0.89, 1.14) per 10% increment | 0.89 |
|  | Percentage of participants with prior radiotherapy | 9 | 3,049 | 0.64 (0.24, 1.74) per 10% increment | 0.35 |
|  | Percentage of participants in ECOG>=1 | 11 | 4,503 | 1.09 (0.73, 1.64) per 10% increment | 0.65 |
|  | Jadad scale of study quality | 14 | 5,841 | 1.39 (0.80, 2.41) per 1 point increment | 0.22 |
