# Supplementary figures and images for: Systematic Review of the Risk of Adverse Outcomes Associated with Vascular Endothelial Growth Factor Inhibitors for the Treatment of Cancer
Source: PLoS One. 2014 Jul 2;9(7):e101145. doi: 10.1371/journal.pone.0101145 (PMC4079504; doi:10.1371/journal.pone.0101145)

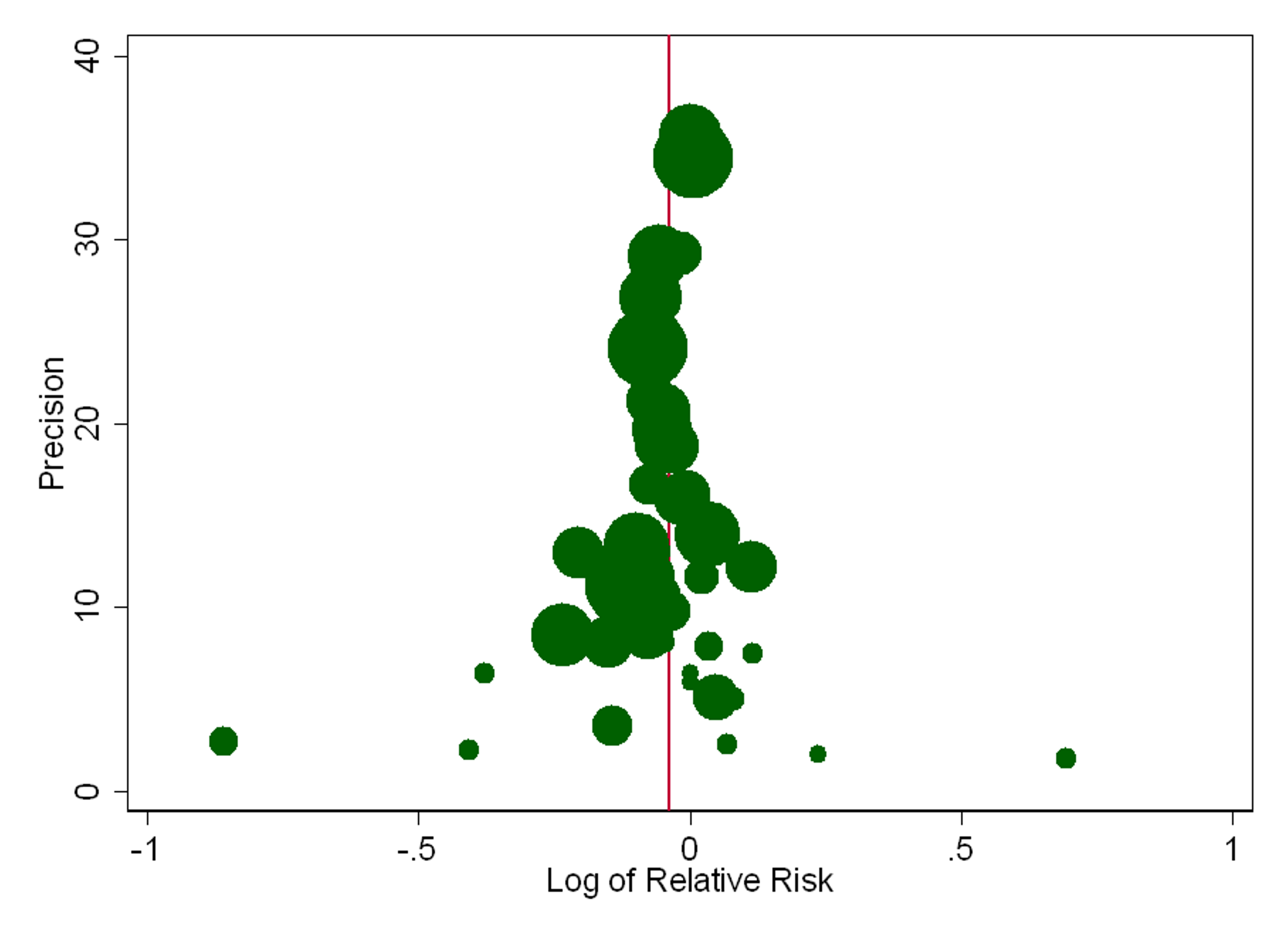

Supplement: eFigure S1 — Publication bias assessment for all-cause mortality. Funnel plot for all-cause death. No funnel plot asymmetry (bias = −0.34, p = 0.20). Our funnel plot appears symmetric. Therefore, there are unlikely to be missing studies favouring either VEGFi or no VEGFi treatment. (TIF) [file pone.0101145.s006.tif]
