# Supplementary material for: Systematic Review of the Risk of Adverse Outcomes Associated with Vascular Endothelial Growth Factor Inhibitors for the Treatment of Cancer
Source: PLoS One. 2014 Jul 2;9(7):e101145. doi: 10.1371/journal.pone.0101145 (PMC4079504; doi:10.1371/journal.pone.0101145)
Supplement: eReference S1 — References of included trials. (DOC) [file pone.0101145.s007.doc]

**eReference S1. References of included trials**

1. Kabbinavar F, Hurwitz HI, Fehrenbacher L, Meropol NJ, Novotny WF, et al. (2003) Phase II, randomized trial comparing bevacizumab plus fluorouracil (FU)/leucovorin (LV) with FU/LV alone in patients with metastatic colorectal cancer.[see comment]. Journal of Clinical Oncology 21: 60-65.
2. Yang JC, Haworth L, Sherry RM, Hwu P, Schwartzentruber DJ, et al. (2003) A randomized trial of bevacizumab, an anti-vascular endothelial growth factor antibody, for metastatic renal cancer. New England Journal of Medicine 349: 427-434.
3. Hurwitz H, Fehrenbacher L, Novotny W, Cartwright T, Hainsworth J, et al. (2004) Bevacizumab plus irinotecan, fluorouracil, and leucovorin for metastatic colorectal cancer. New England Journal of Medicine 350: 2335-2342.
4. Johnson DH, Fehrenbacher L, Novotny WF, Herbst RS, Nemunaitis JJ, et al. (2004) Randomized phase II trial comparing bevacizumab plus carboplatin and paclitaxel with carboplatin and paclitaxel alone in previously untreated locally advanced or metastatic non-small-cell lung cancer. Journal of Clinical Oncology 22: 2184-2191.
5. Kabbinavar FF, Schulz J, McCleod M, Patel T, Hamm JT, et al. (2005) Addition of bevacizumab to bolus fluorouracil and leucovorin in first-line metastatic colorectal cancer: results of a randomized phase II trial.[see comment]. Journal of Clinical Oncology 23: 3697-3705.
6. Miller KD, Chap LI, Holmes FA, Cobleigh MA, Marcom PK, et al. (2005) Randomized phase III trial of capecitabine compared with bevacizumab plus capecitabine in patients with previously treated metastatic breast cancer. Journal of Clinical Oncology 23: 792-799.
7. Demetri GD, van Oosterom AT, Garrett CR, Blackstein ME, Shah MH, et al. (2006) Efficacy and safety of sunitinib in patients with advanced gastrointestinal stromal tumour after failure of imatinib: a randomised controlled trial.[see comment]. Lancet 368: 1329-1338.
8. Ratain MJ, Eisen T, Stadler WM, Flaherty KT, Kaye SB, et al. (2006) Phase II placebo-controlled randomized discontinuation trial of sorafenib in patients with metastatic renal cell carcinoma. Journal of Clinical Oncology 24: 2505-2512.
9. Sandler A, Gray R, Perry MC, Brahmer J, Schiller JH, et al. (2006) Paclitaxel-carboplatin alone or with bevacizumab for non-small-cell lung cancer.[see comment][erratum appears in N Engl J Med. 2007 Jan 18;356(3):318]. New England Journal of Medicine 355: 2542-2550.
10. Arnold AM, Seymour L, Smylie M, Ding K, Ung Y, et al. (2007) Phase II study of vandetanib or placebo in small-cell lung cancer patients after complete or partial response to induction chemotherapy with or without radiation therapy: National Cancer Institute of Canada Clinical Trials Group Study BR.20. Journal of Clinical Oncology 25: 4278-4284.
11. Cohen MH, Gootenberg J, Keegan P, Pazdur R (2007) FDA drug approval summary: Bevacizumab (Avastin) plus carboplatin and paclitaxel as first-line treatment of advanced/metastatic recurrent nonsquamous non-small cell lung cancer. Oncologist 12: 713-718.
12. Escudier B, Choueiri TK, Oudard S, Szczylik C, Negrier S, et al. (2007) Prognostic factors of metastatic renal cell carcinoma after failure of immunotherapy: new paradigm from a large phase III trial with shark cartilage extract AE 941. Journal of Urology 178: 1901-1905.
13. Escudier B, Eisen T, Stadler WM, Szczylik C, Oudard S, et al. (2007) Sorafenib in advanced clear-cell renal-cell carcinoma.[see comment][erratum appears in N Engl J Med. 2007 Jul 12;357(2):203]. New England Journal of Medicine 356: 125-134.
14. Giantonio BJ, Catalano PJ, Meropol NJ, O'Dwyer PJ, Mitchell EP, et al. (2007) Bevacizumab in combination with oxaliplatin, fluorouracil, and leucovorin (FOLFOX4) for previously treated metastatic colorectal cancer: results from the Eastern Cooperative Oncology Group Study E3200. Journal of Clinical Oncology 25: 1539-1544.
15. Herbst RS, O'Neill VJ, Fehrenbacher L, Belani CP, Bonomi PD, et al. (2007) Phase II study of efficacy and safety of bevacizumab in combination with chemotherapy or erlotinib compared with chemotherapy alone for treatment of recurrent or refractory non small-cell lung cancer. Journal of Clinical Oncology 25: 4743-4750.
16. Heymach JV, Johnson BE, Prager D, Csada E, Roubec J, et al. (2007) Randomized, placebo-controlled phase II study of vandetanib plus docetaxel in previously treated non-small-cell lung cancer. Journal of Clinical Oncology 25: 4270-4277.
17. **Karrison T, Kindler HL, Gandara DR, Lu C, Guterz TL, et al. (2007)** Final analysis of a multi-center, double-blind, placebo-controlled, randomized phase II trial of gemcitabine/cisplatin (GC) plus bevacizumab (B) or placebo (P) in patients (pts) with malignant mesothelioma (MM). Journal of Clinical Oncology 25 (18S): 7526
18. Mao S, Daliani DD, Wang X, Thall PF, Do KA, et al. (2007) Employing the treatment-free interval of intermittent androgen ablation to screen candidate prostate cancer therapies. Prostate 67: 1677-1685.
19. Miller K, Wang M, Gralow J, Dickler M, Cobleigh M, et al. (2007) Paclitaxel plus bevacizumab versus paclitaxel alone for metastatic breast cancer. New England Journal of Medicine 357:2666-2676.
20. Heymach JV, Paz-Ares L, De Braud F, Sebastian M, Stewart DJ, et al. (2008) Randomized phase II study of vandetanib alone or with paclitaxel and carboplatin as first-line treatment for advanced non-small-cell lung cancer. Journal of Clinical Oncology 26: 5407-5415.
21. Llovet JM, Ricci S, Mazzaferro V, Hilgard P, Gane E, et al. (2008) Sorafenib in advanced hepatocellular carcinoma. New England Journal of Medicine 359: 378-390.
22. McDermott DF, Sosman JA, Gonzalez R, Hodi FS, Linette GP, et al. (2008) Double-blind randomized phase II study of the combination of sorafenib and dacarbazine in patients with advanced melanoma: a report from the 11715 Study Group. Journal of Clinical Oncology 26: 2178-2185.
23. Saltz LB, Clarke S, Diaz-Rubio E, Scheithauer W, Figer A, et al. (2008) Bevacizumab in combination with oxaliplatin-based chemotherapy as first-line therapy in metastatic colorectal cancer: A randomized phase III study. Journal of Clinical Oncology 26: 2013-2019.
24. Spano J-P, Chodkiewicz C, Maurel J, Wong R, Wasan H, et al. (2008) Efficacy of gemcitabine plus axitinib compared with gemcitabine alone in patients with advanced pancreatic cancer: an open-label randomised phase II study. Lancet 371: 2101-2108.
25. Allegra CJ, Yothers G, O'Connell MJ, Sharif S, Colangelo LH, et al. (2009) Initial Safety Report of NSABP C-08: A Randomized Phase III Study of Modified FOLFOX6 with or Without Bevacizumab for the Adjuvant Treatment of Patients with Stage II or III Colon Cancer. Journal of Clinical Oncology 27: 3385-3390.
26. Cheng AL, Kang YK, Chen Z, Tsao CJ, Qin S, et al. (2009) Efficacy and safety of sorafenib in patients in the Asia-Pacific region with advanced hepatocellular carcinoma: a phase III randomised, double-blind, placebo-controlled trial. The Lancet Oncology 10: 25-34.
27. Hauschild A, Agarwala SS, Trefzer U, Hogg D, Robert C, et al. (2009) Results of a phase III, randomized, placebo-controlled study of sorafenib in combination with carboplatin and paclitaxel as second-line treatment in patients with unresectable stage III or stage IV melanoma. Journal of Clinical Oncology 27: 2823-2830.
28. Horti J, Widmark A, Stenzl A, Federico MH, Abratt RP, et al. (2009) A randomized, double-blind, placebo-controlled phase II study of vandetanib plus docetaxel/prednisolone in patients with hormone-refractory prostate cancer. Cancer Biotherapy and Radiopharmaceuticals 24: 175-180.
29. Van Cutsem E, Vervenne WL, Bennouna J, Humblet Y, Gill S, et al. (2009) Phase III trial of bevacizumab in combination with gemcitabine and erlotinib in patients with metastatic pancreatic cancer. Journal of Clinical Oncology 27: 2231-2237.
30. Abou-Alfa GK, Johnson P, Knox JJ, Capanu M, Davidenko I, et al. (2010) Doxorubicin plus sorafenib vs doxorubicin alone in patients with advanced hepatocellular carcinoma: A randomized trial. JAMA - Journal of the American Medical Association 304: 2154-2160.
31. Crown J, Dieras V, Staroslawska E, Yardley DA, Davidson N, et al. (2010) Phase III trial of sunitinib (SU) in combination with capecitabine (C) versus C in previously treated advanced breast cancer (ABC). Journal of Clinical Oncology 28 (18): LBA1011
32. Escudier B, Bellmunt J, Negrier S, Bajetta E, Melichar B, et al. (2010) Phase III trial of bevacizumab plus interferon alfa-2a in patients with metastatic renal cell carcinoma (AVOREN): final analysis of overall survival. Journal of Clinical Oncology 28: 2144-2150.
33. Goss GD, Arnold A, Shepherd FA, Dediu M, Ciuleanu T-E, et al. (2010) Randomized, double-blind trial of carboplatin and paclitaxel with either daily oral cediranib or placebo in advanced non-small-cell lung cancer: NCIC clinical trials group BR24 study. Journal of Clinical Oncology 28: 49-55.
34. Herbst RS, Sun Y, Eberhardt WEE, Germonpre P, Saijo N, et al. (2010) Vandetanib plus docetaxel versus docetaxel as second-line treatment for patients with advanced non-small-cell lung cancer (ZODIAC): A double-blind, randomised, phase 3 trial. The Lancet Oncology 11 (7): 619-626.
35. Kemeny NE, Jarnagin WR, Capanu M, Fong Y, Gewirtz AN, et al. (2011) Randomized phase II trial of adjuvant hepatic arterial infusion and systemic chemotherapy with or without bevacizumab in patients with resected hepatic metastases from colorectal cancer. Journal of Clinical Oncology 29: 884-889.
36. Kindler HL, Niedzwiecki D, Hollis D, Sutherland S, Schrag D, et al. (2010) Gemcitabine plus bevacizumab compared with gemcitabine plus placebo in patients with advanced pancreatic cancer: phase III trial of the Cancer and Leukemia Group B (CALGB 80303). Journal of Clinical Oncology 28: 3617-3622.
37. Lu C, Lee JJ, Komaki R, Herbst RS, Feng L, et al. (2010) Chemoradiotherapy with or without AE-941 in stage III non-small cell lung cancer: a randomized phase III trial. Journal of the National Cancer Institute 102: 859-865.
38. Miles DW, Chan A, Dirix LY, Cortes J, Pivot X, et al. (2010) Phase III study of bevacizumab plus docetaxel compared with placebo plus docetaxel for the first-line treatment of human epidermal growth factor receptor 2-negative metastatic breast cancer. Journal of Clinical Oncology 28 (20): 3239-3247.
39. Monk BJ, Mas Lopez L, Zarba JJ, Oaknin A, Tarpin C, et al. (2010) Phase II, open-label study of pazopanib or lapatinib monotherapy compared with pazopanib plus lapatinib combination therapy in patients with advanced and recurrent cervical cancer. Journal of Clinical Oncology 28: 3562-3569.
40. Reck M, von Pawel J, Zatloukal P, Ramlau R, Gorbounova V, et al. (2010) Overall survival with cisplatin-gemcitabine and bevacizumab or placebo as first-line therapy for nonsquamous non-small-cell lung cancer: results from a randomised phase III trial (AVAiL). Annals of Oncology 21: 1804-1809.
41. Rini BI, Halabi S, Rosenberg JE, Stadler WM, Vaena DA, et al. (2010) Phase III trial of bevacizumab plus interferon alfa versus interferon alfa monotherapy in patients with metastatic renal cell carcinoma: final results of CALGB 90206. Journal of Clinical Oncology 28: 2137-2143.
42. Scagliotti G, Novello S, von Pawel J, Reck M, Pereira JR, et al. (2010) Phase III study of carboplatin and paclitaxel alone or with sorafenib in advanced non-small-cell lung cancer. Journal of Clinical Oncology 28: 1835-1842.
43. Serve H, Wagner R, Sauerland C, Brunnberg U, Krug U, et al. (2010) Sorafenib In Combination with Standard Induction and Consolidation Therapy In Elderly AML Patients: Results From a Randomized, Placebo-Controlled Phase II Trial. Blood 116: 151.
44. Stathopoulos GP, Batziou C, Trafalis D, Koutantos J, Batzios S, et al. (2010) Treatment of colorectal cancer with and without bevacizumab: a phase III study. Oncology 78: 376-381.
45. Sternberg CN, Davis ID, Mardiak J, Szczylik C, Lee E, et al. (2010) Pazopanib in locally advanced or metastatic renal cell carcinoma: results of a randomized phase III trial. Journal of Clinical Oncology 28: 1061-1068.
46. Tebbutt NC, Wilson K, Gebski VJ, Cummins MM, Zannino D, et al. (2010) Capecitabine, bevacizumab, and mitomycin in first-line treatment of metastatic colorectal cancer: results of the Australasian Gastrointestinal Trials Group Randomized Phase III MAX Study. Journal of Clinical Oncology 28: 3191-3198.
47. Brufsky AM, Hurvitz S, Perez E, Swamy R, Valero V, et al. (2011) RIBBON-2: a randomized, double-blind, placebo-controlled, phase III trial evaluating the efficacy and safety of bevacizumab in combination with chemotherapy for second-line treatment of human epidermal growth factor receptor 2-negative metastatic breast cancer. Journal of Clinical Oncology 29: 4286-4293.
48. Burger RA, Brady MF, Bookman MA, Fleming GF, Monk BJ, et al. (2011) Incorporation of bevacizumab in the primary treatment of ovarian cancer. New England Journal of Medicine 365: 2473-2483.
49. Choueiri TK, Ross RW, Jacobus S, Vaishampayan U, Yu EY, et al. (2012) Double-blind, randomized trial of docetaxel plus vandetanib versus docetaxel plus placebo in platinum-pretreated metastatic urothelial cancer. Journal of Clinical Oncology 30: 507-512. Published online ahead of print at www.jco.org on December 19, 2011.
50. de Boer RH, Arrieta O, Yang C-H, Gottfried M, Chan V, et al. (2011) Vandetanib plus pemetrexed for the second-line treatment of advanced non-small-cell lung cancer: a randomized, double-blind phase III trial. Journal of Clinical Oncology 29: 1067-1074.
51. Guan ZZ, Xu JM, Luo RC, Feng FY, Wang LW, et al. (2011) Efficacy and safety of bevacizumab plus chemotherapy in chinese patients with metastatic colorectal cancer:A randomized phase iii artist trial. Chinese Journal of Cancer 30 (10): 682-689.
52. Hecht JR, Trarbach T, Hainsworth JD, Major P, Jager E, et al. (2011) Randomized, placebo-controlled, phase III study of first-line oxaliplatin-based chemotherapy plus PTK787/ZK 222584, an oral vascular endothelial growth factor receptor inhibitor, in patients with metastatic colorectal adenocarcinoma. Journal of Clinical Oncology 29: 1997-2003.
53. Herbst RS, Ansari R, Bustin F, Flynn P, Hart L, et al. (2011) Efficacy of bevacizumab plus erlotinib versus erlotinib alone in advanced non-small-cell lung cancer after failure of standard first-line chemotherapy (BeTa): a double-blind, placebo-controlled, phase 3 trial. Lancet 377: 1846-1854.
54. Kato T, Muro K, Yamaguchi K, Bando H, Hazama S, et al. (2012) Cediranib in combination with mFOLFOX6 in Japanese patients with metastatic colorectal cancer: Results from the randomised phase II part of a phase I/II study. Annals of Oncology 23: 933-941. Published online 9 August 2011
55. Kim KB, Sosman JA, Fruehauf JP, Linette GP, Markovic SN, et al. (2012) BEAM: A randomized phase II study evaluating the activity of bevacizumab in combination with carboplatin plus paclitaxel in patients with previously untreated advanced melanoma. Journal of Clinical Oncology 30 (1): 34-41. Published online ahead of print at www.jco.org on November 28, 2011.
56. Kindler HL, Ioka T, Richel DJ, Bennouna J, Letourneau R, et al. (2011) Axitinib plus gemcitabine versus placebo plus gemcitabine in patients with advanced pancreatic adenocarcinoma: A double-blind randomised phase 3 study. The Lancet Oncology 12 (3): 256-262.
57. Kudo M, Imanaka K, Chida N, Nakachi K, Tak WY, et al. (2011) Phase III study of sorafenib after transarterial chemoembolisation in Japanese and Korean patients with unresectable hepatocellular carcinoma. European Journal of Cancer 47: 2117-2127.
58. Loriot Y, Houede N, Le Moulec S, Hennequin C, Eymard JC, et al. (2011) Bicalutamide in Combination With Vandetanib or Placebo in Patients With Castration-refractory Metastatic Prostate Cancer Without Any Clinical Symptom Related to Disease Progression - a Randomized, Double-blind Phase II Trial. European Journal of Cancer 47: S500.
59. Martin M, Roche H, Pinter T, Crown J, Kennedy MJ, et al. (2011) Motesanib, or open-label bevacizumab, in combination with paclitaxel, as first-line treatment for HER2-negative locally recurrent or metastatic breast cancer: a phase 2, randomised, double-blind, placebo-controlled study.[Erratum appears in Lancet Oncol. 2011 Aug;12(8):722]. Lancet Oncology 12: 369-376.
60. Ohtsu A, Shah MA, Van Cutsem E, Rha SY, Sawaki A, et al. (2011) Bevacizumab in combination with chemotherapy as first-line therapy in advanced gastric cancer: a randomized, double-blind, placebo-controlled phase III study. Journal of Clinical Oncology 29: 3968-3976.
61. Perren TJ, Swart AM, Pfisterer J, Ledermann JA, Pujade-Lauraine E, et al. (2011) A phase 3 trial of bevacizumab in ovarian cancer. New England Journal of Medicine 365: 2484-2496.
62. Raymond E, Dahan L, Raoul J-L, Bang Y-J, Borbath I, et al. (2011) Sunitinib malate for the treatment of pancreatic neuroendocrine tumors.[Erratum appears in N Engl J Med. 2011 Mar 17;364(11):1082]. New England Journal of Medicine 364: 501-513.
63. Robert NJ, Dieras V, Glaspy J, Brufsky AM, Bondarenko I, et al. (2011) RIBBON-1: randomized, double-blind, placebo-controlled, phase III trial of chemotherapy with or without bevacizumab for first-line treatment of human epidermal growth factor receptor 2-negative, locally recurrent or metastatic breast cancer. Journal of Clinical Oncology 29: 1252-1260.
64. Rugo HS, Stopeck AT, Joy AA, Chan S, Verma S, et al. (2011) Randomized, placebo-controlled, double-blind, phase II study of axitinib plus docetaxel versus docetaxel plus placebo in patients with metastatic breast cancer. Journal of Clinical Oncology 29: 2459-2465.
65. Spigel DR, Townley PM, Waterhouse DM, Fang L, Adiguzel I, et al. (2011) Randomized phase II study of bevacizumab in combination with chemotherapy in previously untreated extensive-stage small-cell lung cancer: results from the SALUTE trial. Journal of Clinical Oncology 29: 2215-2222.
66. Spigel DR, Burris IHA, Greco FA, Shipley DL, Friedman EK, et al. (2011) Randomized, double-blind, placebo-controlled, phase II trial of sorafenib and erlotinib or erlotinib alone in previously treated advanced non-small-cell lung cancer. Journal of Clinical Oncology 29 (18): 2582-2589.
67. Van Cutsem E, Bajetta E, Valle J, Kohne C-H, Hecht JR, et al. (2011) Randomized, placebo-controlled, phase III study of oxaliplatin, fluorouracil, and leucovorin with or without PTK787/ZK 222584 in patients with previously treated metastatic colorectal adenocarcinoma. Journal of Clinical Oncology 29: 2004-2010.
68. Wells Jr SA, Robinson BG, Gagel RF, Dralle H, Fagin JA, et al. (2012) Vandetanib in patients with locally advanced or metastatic medullary thyroid cancer: A randomized, double-blind phase III trial. Journal of Clinical Oncology 30 (2): 134-141. Published online ahead of print at www.jco.org on October 24, 2011.
69. Yang Y, Lu Y, Wang C, Qu J, Chang X, et al. (2011) Efficacy and safety of sorafenib and cryotherapy for advanced hepatocellular carcinoma (hcc): a single center randomized study. Hepatology 54: 1367A.
70. Bear HD, Tang G, Rastogi P, Geyer Jr CE, Robidoux A, et al. (2012) Bevacizumab added to neoadjuvant chemotherapy for breast cancer. New England Journal of Medicine 366 (4): 310-320.
71. Kelly WK, Halabi S, Carducci M, George D, Mahoney JF, et al. (2012) Randomized, Double-Blind, Placebo-Controlled Phase III Trial Comparing Docetaxel and Prednisone With or Without Bevacizumab in Men With Metastatic Castration-Resistant Prostate Cancer: CALGB 90401 J Clin Oncol 30:1534-1540.
72. Von Minckwitz G, Eidtmann H, Rezai M, Fasching PA, Tesch H, et al. (2012) Neoadjuvant chemotherapy and bevacizumab for HER2-negative breast cancer. New England Journal of Medicine 366 (4): 299-309.
